# Hiding deep in the trees: discovery of divergent mitochondrial lineages in Malagasy chameleons of the *Calumma nasutum* group

**DOI:** 10.1002/ece3.269

**Published:** 2012-07

**Authors:** Philip-Sebastian Gehring, Krystal A Tolley, Falk Sebastian Eckhardt, Ted M Townsend, Thomas Ziegler, Fanomezana Ratsoavina, Frank Glaw, Miguel Vences

**Affiliations:** 1 Zoological Institute, Technical University of BraunschweigMendelssohnstr. 4, 38106 Braunschweig, Germany; 2 Applied Biodiversity Research Division, South African National Biodiversity InstituteClaremont 7735, Cape Town, South Africa; 3 Department of Botany and Zoology, University of StellenboschMatieland 7602, South Africa; 4 Tierärztliche Hochschule Hannover, Zoological InstituteBünteweg 17, 30559 Hannover, Germany; 5 Department of Biology, San Diego State UniversitySan Diego, California 92182; 6 Cologne ZooRiehler Straße 173, 50735 Köln, Germany; 7 Département de Biologie Animale, Université d’AntananarivoBP 906, Antananarivo 101, Madagascar; 8 Zoologische Staatssammlung MünchenMünchhausenstr. 21, 81247 München, Germany

**Keywords:** *Calumma nasutum* group, Chamaeleonidae, genital morphology, Madagascar, molecular diversity, snout appendage scales

## Abstract

We conducted a comprehensive molecular phylogenetic study for a group of chameleons from Madagascar (Chamaeleonidae: *Calumma nasutum* group, comprising seven nominal species) to examine the genetic and species diversity in this widespread genus. Based on DNA sequences of the mitochondrial gene (*ND2*) from 215 specimens, we reconstructed the phylogeny using a Bayesian approach. Our results show deep divergences among several unnamed mitochondrial lineages that are difficult to identify morphologically. We evaluated lineage diversification using a number of statistical phylogenetic methods (general mixed Yule-coalescent model; SpeciesIdentifier; net *p*-distances) to objectively delimit lineages that we here consider as operational taxonomic units (OTUs), and for which the taxonomic status remains largely unknown. In addition, we compared molecular and morphological differentiation in detail for one particularly diverse clade (the *C. boettgeri* complex) from northern Madagascar. To assess the species boundaries within this group we used an integrative taxonomic approach, combining evidence from two independent molecular markers (*ND2* and *CMOS*), together with genital and other external morphological characters, and conclude that some of the newly discovered OTUs are separate species (confirmed candidate species, CCS), while others should best be considered as deep conspecific lineages (DCLs). Our analysis supports a total of 33 OTUs, of which seven correspond to described species, suggesting that the taxonomy of the *C. nasutum* group is in need of revision.

## Introduction

Accurately inventorying and delimiting species is a fundamental prerequisite for numerous fields of biological research, such as evolutionary theory, biogeography, and conservation planning (e.g., Mayr, 1997). Despite this importance, biologists are still far from possessing a complete species inventory of our planet, which in turn seriously hampers our ability to conserve biotic resources (UNEP, 2008). Even within vertebrates, one of the best-known groups of organisms, there are hundreds if not thousands of species yet to be described (e.g., MacKinnon, 2000; Meegaskumbura et al., 2002; Fouquet et al., 2007; Vieites et al., 2009). The largest proportion of undescribed biotic diversity is suspected to live in the tropics, and one megadiverse area still containing many undescribed species is Madagascar. This, the globe's fourth largest island, harbors one of the most spectacular but threatened biotas, with many components such as the amphibians and reptiles characterized by an extreme degree of endemicity. Madagascar's herpetofauna has been more intensively studied in the past two decades compared to most tropical areas, yet this scrutiny has paradoxically revealed an enormous gap in taxonomic knowledge, and many species still await formal description.

While the largest numbers of undescribed species have been identified among amphibians (Köhler et al., 2005; Vieites et al., 2009), recent comprehensive taxonomic reviews have also revealed that numerous widespread Malagasy reptile species actually represent species complexes (e.g., Raxworthy and Nussbaum, 2006; Gehring et al., 2010, 2011; Crottini et al., 2011; Miralles et al., 2011). Although the phenomenon of morphologically cryptic species has been recognized by taxonomists for nearly 300 years (Bickford et al., 2007), it remains largely unexplored among Malagasy reptiles.

The ease of routinely sequencing short DNA fragments to identify candidate species (DNA barcoding) has greatly facilitated species inventories. However, this represents only the first step in integrative taxonomic practice (Padial et al., 2010), and its uncritical use can lead to poor-quality species delimitation because deeply divergent alleles can coexist within species due to introgression and incomplete lineage sorting, resulting in apparent species paraphyly in mitochondrial DNA (mtDNA; Funk and Omland, 2003). Hence, the finding of unexpected genetic diversity in some Malagasy vertebrates and its translation into species description has led to doubts whether current practice represents taxonomic inflation, or is a true representation of species diversity (e.g., Isaac et al., 2004; Tattersall, 2007; Weisrock et al., 2010). For example, the species diversity of mouse lemurs (genus *Microcebus*) has increased more than sevenfold during the last two decades, largely through the analysis of mtDNA sequence data only (Tattersall, 2007; Weisrock et al., 2010).

**Figure 1:**
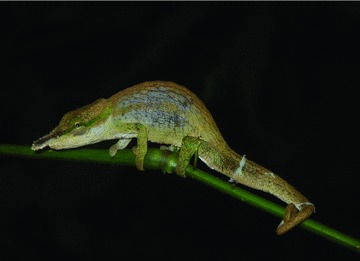
Male specimen of *Calumma guibei* from the Tsaratanana Massif, Madagascar, in life. Note the rostral appendage that is flexible and dermal (no underlying bony structure) in all species of the *C. nasutum* group, and the small occipital lobes which are characteristic for the species of the *C. boettgeri* complex.

Taxonomic progress toward identification and description of new species leads to a further challenge in biodiversity studies and conservation planning: once a formerly widespread species is split into several new species, historical distribution records become unreliable with respect to the new taxonomy, which subsequently leads to decreased reliability in the biogeographic data available. For example, the splitting of the Malagasy chameleon *Calumma brevicorne* into several new species (Raxworthy and Nussbaum, 2006) resulted in reliable records existing from only a few localities per species (Glaw and Vences, 2007). The same situation may hold true for the chameleons in the *C. nasutum* group, which is presumed to include six or seven species, widely distributed across the rainforests in eastern and northern Madagascar. All of these species are easily identified as members of the group by morphology (most obviously by the soft dermal appendages on their snout tips; Fig. 1), and most of them can also be diagnosed to species with some confidence following current taxonomy (see Gehring et al., 2011). However, some species such as *C. nasutum* are known to harbor important morphological variation between and within populations (e.g., absence or presence of dorsal crests, shape, length, and coloration of rostral appendages), suggesting a potentially complex taxonomic situation (e.g., Hillenius, 1959; Brygoo, 1971; Glaw and Vences, 2007; Gehring et al., 2011).

The aim of the present study is a preliminary assessment of genetic diversity among these small-sized chameleons. To test the hypothesis that previously undiscovered deep genetic lineages are present, we (1) produced a phylogeny from a fragment of the mitochondrial *ND2* gene from 215 samples covering the entire distribution of all species, (2) applied divergence thresholds using sequence divergence estimates, (3) applied a coalescence-based method of species delimitation (general mixed Yule-coalescent model [GMYC]) to compare sequence divergences and subsequently generate candidate species hypotheses, and (4) performed preliminary tests of the candidate species hypotheses in one morphologically well-delimited complex of these chameleons from northern Madagascar (the *C. boettgeri* complex) based on the integration of evidence from mtDNA and nucDNA sequences, distribution patterns (i.e., occurrence of syntopy), genital morphology, and pholidosis.

## Materials and Methods

### Taxonomy and terminology

To facilitate the presentation of results we herein use a number of predefined terms to refer to units in our phylogenetic trees and assemblages of taxa. First, we follow previous authors, in particular Brygoo (1971), who defined phenetic species groups among Malagasy chameleons, and apply the term “*Calumma nasutum* group” to all species in the genus *Calumma* Gray, 1865 with flexible (“nose” like) rostral appendages. Species-level taxonomy of these chameleons herein follows Gehring et al. (2011), Raxworthy et al. (2008), and Andreone et al. (2001), according to which the group contains the nominal taxa *C. boettgeri* (Boulenger, 1888), *C. fallax* (Mocquard, 1900), *C. gallus* (Günther, 1877), *C. guibei* (Hillenius, 1959; Fig. 1), *C. linotum* (Müller, 1924), *C. nasutum* (Duméril and Bibron, 1836), and *C. vohibola* (Gehring et al., 2011). Within the *C. nasutum* group, three species from northern Madagascar (*C. boettgeri*, *C. guibei*, *C. linotum*) are easily and immediately recognized by having well-defined occipital lobes, although it is uncertain whether these species form a monophyletic group. We refer to these three species herein as the *C. boettgeri* complex.

The species delimitation approaches used herein are based on a phylogenetic tree derived from an mtDNA gene, and species hypotheses were produced by drawing species limits at certain nodes of the tree. We refer to genealogical units at or below the species level as lineages. Those lineages supported by the species delimitation approaches (see below) are here termed operational taxonomic units (OTUs). For a subset of species, additional evidence from morphology or nuclear genes was available; following Vieites et al. (2009) we use the terms confirmed candidate species (CCS) for lineages supported by at least two independent lines of evidence, and deep conspecific lineage (DCL) if the available evidence rejects evolutionary independence and thus species status.

Some of the lineages identified are morphologically similar and are grouped phylogenetically to form a number of well-supported major clades. Furthermore we use the term “cluster” in some cases to refer to the results of some of the species delimitation approaches which are based on clustering sequences on the basis of their similarity (see below).

### Sample collection and morphology

Specimens were collected in the field largely at night using torches and headlamps to detect roosting chameleons in the vegetation. Voucher specimens were anesthetized and euthanized, fixed with 90% ethanol and stored in 70% ethanol. Muscle tissue samples for molecular analyses were taken from the tongue, hind limb, or tail and preserved in pure ethanol. Individuals that were not collected as vouchers were sampled and released. All tissue samples were stored in 99% ethanol. ZCMV, FGZC, DRV, MPFC, ACZC, ZCSH, and PSG refer to field numbers of M. Vences, F. Glaw, D. Vieites, M. Pabijan, A. Crottini, S. Hauswaldt, and P.-S. Gehring, respectively. Representative voucher specimens were collected and deposited in the herpetological collections of the Museo Nacional de Ciencias Naturales (MNCN), Zoologisches Forschungsmuseum Alexander Koenig, Bonn (ZFMK), Zoölogisch Museum Amsterdam (ZMA), Zoologische Staatssammlung München (ZSM), and the Université d’Antananarivo, Département de Biologie Animale (UADBA). Geographic coordinates were recorded with GPS receivers. (Table S1 provides detailed information on GPS coordinates of sampling locations.) A distribution map including an elevation layer of sample localities in Madagascar was obtained using the program DIVA GIS (Version 7.3.0.1; http://diva-gis.org). The taxonomy used herein follows Raxworthy et al. (2008) for the definition of *C. boettgeri*, *C. guibei*, and *C. linotum*, and Glaw and Vences (2007) and Gehring et al. (2011) for *C. fallax*, *C. nasutum*, and *C. vohibola.* Samples of *C. oshaughnessyi* (FGZC 4577) and *Brookesia ramanantsoai* (FGZC 4557) were used as outgroups in the molecular analyses.

For study of hemipenes, these structures were everted directly after euthanasia, by first injecting water and subsequently fixative into the organs. Hemipenes were examined under a stereo microscope and hand drawn. Variation and possible artifacts caused especially by incomplete eversion of organs or detailed structures were assessed by examining multiple specimens per lineage in the *C. boettgeri* complex. Terminology of hemipenial structures follows Klaver and Böhme (1986) and Böhme (1988).

### Laboratory protocols

Total genomic DNA was extracted from the tissue samples using proteinase K digestion (10 mg/mL concentration) followed by a salt extraction protocol (Bruford et al., 1992). A fragment of the mitochondrial gene for NADH dehydrogenase subunit 2 (*ND2*) was PCR-amplified using the primers ND2F17 (5′-TGACAAAAAATTGCNCC-3′) (Macey et al., 2000) and ALAR2 (5′-AAAATRTCTGRGTTGCATTCAG-3′) (Macey et al., 1997), and a fragment of the nuclear DNA (nucDNA) from the oocyte maturation factor gene (*CMOS*) using the primers CO8 (5′-GCTTGGTGTTCAATAGACTGG-3′) and CO9 (5′-TTTGGGAGCATCCAAAGTCTC-3′) (Han et al., 2004), following standard protocols. After purification with ExoSAP-IT (USB), the fragments were sequenced with an automated DNA sequencer (ABI 3130 XL Applied Biosystems, Foster City, USA). For the *ND2* dataset only the light strand was sequenced, while the *CMOS* gene fragment was sequenced in both directions to verify possible heterozygotes. Quality of sequence chromatograms was verified and sequences were aligned manually by amino-acid translations with the software CodonCode Aligner (CodonCode Corporation, Dedham, USA). All newly determined sequences were submitted to GenBank (accession numbers JQ733989–JQ734169 and JQ614354–JQ614415; see also Table S1).

### Phylogenetic analysis

Three sets of DNA sequences were used for analyses: (1) a 586 bp fragment of *ND2* from all available samples (215 specimens; 103 unique sequences), (2) a subset of these same *ND2* sequences trimmed to exactly the same length in all taxa (389 bp; no missing data) from only chameleons with flexible rostral appendages as well as occipital lobes (the *C. boettgeri* complex; *N* = 82), and (3) a 500 bp fragment of *CMOS* for most samples of the *C. boettgeri* complex (*N* = 63). The major focus of the present paper is on species delimitation, not on detailed reconstruction of the phylogeny of the *C. nasutum* group, for which the short DNA fragments used herein would be clearly insufficient. We analyzed three separate datasets using multiple complementary methods to examine the hypothesis that deep, potentially species-level divergences exist within the species complex:

1. Phylogenetic analysis of the full *ND2* datasets with each unique sequence represented only once (*N* = 103) was carried out using Bayesian inference in BEAST (Drummond and Rambaut, 2007), in order to obtain an ultrametric tree for automated species delimitation analysis (see section “Species Delimitation” below for details). In order to graphically represent the phylogenetic placement of the sequences of all specimens (*N* = 215) we also ran a Bayesian inference analysis with MrBayes version 3.1.2 (Ronquist and Huelsenbeck, 2003) under the same settings as for the *C. boettgeri* complex data (given in the next paragraph); this tree is only represented in the Supporting information (Fig. S1) and was not used further in the study.
2. Analysis of the subset of *ND2* sequences of only the *C. boettgeri* complex was carried out with MrBayes under substitution models determined with MrModeltest 2.3 (Nylander, 2004) with two independent runs of 20 million generations, started on random trees and each with four incrementally heated Markov chains (using default heating values), sampling the Markov chains at intervals of 1000 generations. Convergence of the Markov chains was checked using Tracer v1.5 (Rambaut and Drummond, 2009) and mixing of chains was assessed with AWTY (Nylander et al., 2008). The first 25% of the trees were conservatively discarded and the trees of both runs retained post burn-in were used to generate a 50% majority rule consensus tree.
3. *CMOS* sequence was used as independent genetic marker to confirm the absence or reduction of gene flow among genealogical lineages initially identified on the basis of the *ND2* analysis. Because haplotype sharing among lineages can also reflect incomplete lineage sorting, this kind of evidence is asymmetric: haplotype sharing in independent markers cannot reject reduction of gene flow but two groups of specimens not sharing haplotypes of multiple independent markers are likely to be on independent evolutionary trajectories without gene flow (the genealogic concordance method of phylogenetic species recognition, proposed by Avise and Ball, 1990). Because we found only minor variation in *CMOS*, we used a haplotype network approach for this gene. Sequences were phased into distinct haplotypes using the Phase algorithm implemented in the software DNASp version 5.10.3 (Librado and Rozas, 2009). A haplotype network was then constructed from the nuclear haplotypes using TCS (Clement et al., 2000). Ambiguities in the network were solved manually using frequency, topological, and geographical criteria (e.g., Posada and Crandall, 2001).

### Species delimitation

To examine the genetic diversity in this group, three approaches were used on the basis of the full set of *ND2* sequences (506 bp, 215 specimens; 103 unique sequences). Of these the approaches 1 and 2 (see below) searched for the breaks in overlap between intra- and interspecific variation, whereas approach 3 was a coalescent-based method that attempts to identify boundaries between species-level and population-level processes from tree shape and branch lengths.

In approach 1, the topology obtained in the Bayesian analysis was used as a framework for manually grouping taxa at different levels (excluding duplicate haplotypes, remaining sequences for analysis *N* = 103) by searching for the threshold at which the intraclade *p*-distances in the *ND2* sequences were lower than the interclade *p*-distances. We performed this approach using net *p*-distances, that is, the average *p*-distances between lineages subtracting the differences within each of the lineages. Taxa were grouped into clades starting at the base of the tree, and moving outward toward the tips until supported nodes (PP of 0.95 or higher) were encountered. The net *p*-distances among and within these deep lineages were then calculated. The taxa were then regrouped into different combinations of lineages by moving outward to the next set of supported nodes and the net *p*-distances estimated again. The procedure was repeated a final time, moving outward to the next set of supported nodes, and *p*-distances again estimated for these shallow lineages.

In approach 2, the SpeciesIdentifier “Cluster” algorithm in Taxon DNA v 1.7 (Meier et al., 2006) was used to automatically cluster taxa according to pairwise distances for every sequence within each cluster. Incremental threshold values of 2–10% were used, whereby the maximum pairwise distance within each cluster (possible species-level lineage) should not exceed a given threshold.

Finally, in approach 3, clustering was examined using a GMYC in which the boundaries between species-level and population-level processes are identified (Pons et al., 2006; Fontaneto et al., 2007). In this method, the transition in branching rates from lineage diversification processes (Yule model) to population-level diversification (coalescent model) are identified. The GMYC model can be employed with a single transition (Pons et al., 2006) or a multiple transition that allows the depth of the coalescent-speciation transition to vary across the phylogenetic tree (Monaghan et al., 2009). The observed branching rate is then compared against a null model that assumes no shift in branching rate using a log-likelihood ratio test (LRT). Failure to reject the null model implies no shift in the branching rate (i.e., a single species is present). An ultrametric tree was created in BEAST (Drummond and Rambaut, 2007), using a Yule model for the tree prior, a strict molecular clock (fixed to a rate of 1.0), a random starting tree, and the GTR+I+G model of sequence evolution. Tracer v. 1.5 (Rambaut and Drummond, 2009) was used to verify that the effective sample size for all estimated parameters exceeded 200. The ultrametric tree was obtained using TreeAnnotator in BEAST (Drummond and Rambaut, 2007) specifying a 10% burn-in, the maximum credibility clade tree, and saving the target node heights. To ensure that the choice of priors in BEAST did not affect the result, the ultrametric tree was also obtained using (1) a Yule model with a lognormal relaxed clock, (2) a coalescent model with a strict clock, and (3) a coalescent model with a log-normal relaxed clock, following Monaghan et al. (2009). The four trees obtained were fit to both single-transition and multiple-transition GMYC models using the Splits and Ape packages in R. The fit of the single and multiple GMYC models were compared with a χ^2^-test in R using the Splits package (Monaghan et al. 2009).

Focusing mainly on the *C. boettgeri* complex, we furthermore followed an integrative taxonomy approach that utilizes the best available evidence from different methods and approaches (Padial et al., 2010). We took (1) syntopic occurrence without genetic admixture and with maintenance of morphological differences, (2) congruent signals from largely independent character sets (morphology, mtDNA, nucDNA), and (3) distinct and constant differences in characters mediating reproductive isolation (i.e., genital morpho-logy) as indications of evolutionary independence and thus species status of lineages.

## Results

### *ND2* phylogeny and sequence diversity

**Figure 2:**
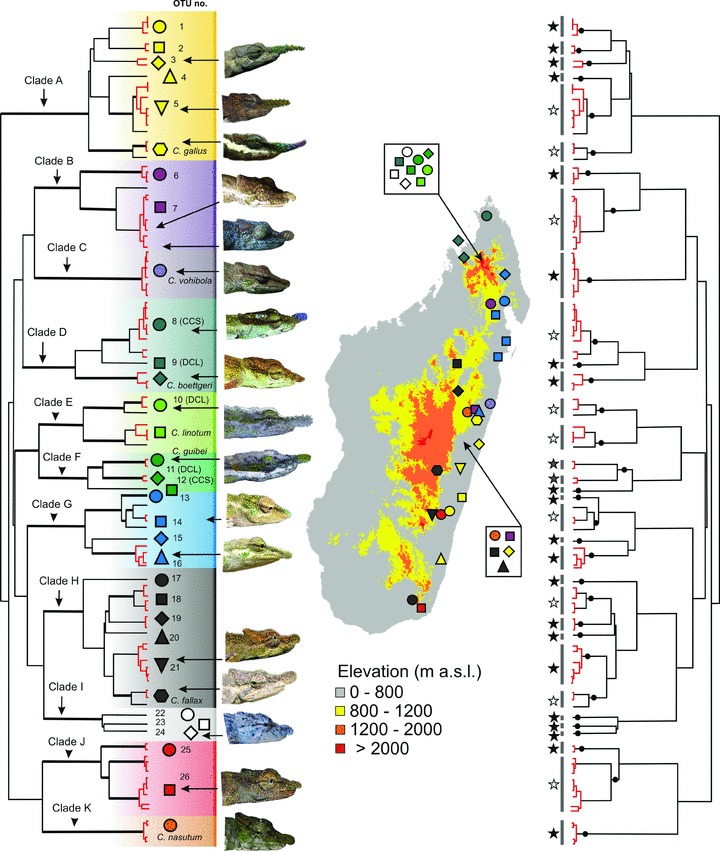
An ultrametric tree for the *ND2* gene fragment of Malagasy chameleons of the *Calumma nasutum* group, using Bayesian inference analysis with BEAST, a Yule model, and a strict clock. *Calumma oshaughnessyi* and *Brookesia ramanantsoai* were used as outgroup taxa (not shown). In the tree on the left Bayesian posterior probability values above a threshold of 0.9 are indicated by broadened lines; strongly supported major clades are indicated in colors and numbered with capital letters. Different symbols characterize lineages that were flagged as candidate species by two of three different methods of species delineation. *Nomina* currently considered as valid are tentatively assigned to these lineages based mainly on their type localities. Locality information for each lineage is given in the map of Madagascar. In the ultrametric tree on the right the lineages are indicated based on three different clustering methods (net *p*-distances: black dots, SpeciesIdentifier: gray bars; and GMYC clusters: branches in red). Black stars denote groups for which all three methods were in agreement, white stars denote groups for which net *p*-distances and SpeciesIdentifier were in agreement, and gray stars indicate groups for which GMYC and SpeciesIdentifier were in agreement.

Altogether, 103 unique *ND2* sequences were obtained from 215 *Calumma* individuals from more than 60 different locations (Fig. S1 and Table S1), and after exclusion of duplicate haplotypes, 103 sequences were used to create the ultrametric tree in BEAST (Fig. 2; a detailed version of this tree is given in Fig. S2).

Based on net *p*-distances for the entire *ND2* dataset (*p*Dist–approach 1; Fig. S1), three sets of possible species-level lineages were identified: (1) 11 lineages with divergences >8.2% between and <8.5% within them; (2) 27 lineages with divergences >6% between and <4.5% within them, and (3) 32 lineages with divergences >6% between and <3% within them (Fig. 2; Table S2). Because the first set of divergences contained an overlap in values for the between and within *p*-distances, this set was considered the least likely to accurately reflect the number of independent evolutionary lineages. Both the second and third sets had gaps in values for the between and within *p-*distances, and in both cases the divergence among lineages was at least 6%. However, for the third set, the within-lineage divergences were smaller (<3%) than those within the second set (<4.5%), resulting in a larger gap for the values within and between lineages.

Using SpeciesIdentifier (SpeciesID—approach 2), the number of species-level lineages identified depended on the threshold used, with the largest threshold producing the fewest lineages (Table S3). Given that approach 1 shows that between-lineage *p*-distances are maximized when the within-lineage value is no more than 4.5%, a threshold of 4% or 5% should produce similar results for approach 2. For these data, the 5% threshold produced 34 lineages, a value similar to that of approach 1.

The number of species-level lineages defined by the GMYC model (approach 3) relied somewhat on the priors used to construct the ultrametric tree, ranging from 44 to 53 OTUs (Table S4). There was no significant difference between the single- and multiple-threshold approaches for any tree models. The single threshold GMYC model utilizing a tree based on a coalescent model and a strict clock produced the least number of clusters (23) but not the fewest singletons (24). Overall, the Yule model with the strict clock as well as the coalescent model with the relaxed clock produced fewer total lineages (44), comprised of 26 clusters and 18 singletons. For further analysis, we applied a conservative approach and identified those lineages that were supported by at least two out of three methods (*p*-distance criteria, SpeciesIdentifier, and GMYC). This yielded a total of 33 OTUs, which we assigned to species complexes based on morphological characters considered to be diagnostic among species as previously defined (mainly presence vs. absence of occipital lobes, and size and shape of rostral appendage) and placement of topotypical populations of some species in the tree (Fig. 2).

### Mitochondrial and nuclear sequence diversity in the *C. boettgeri* complex

**Figure 3:**
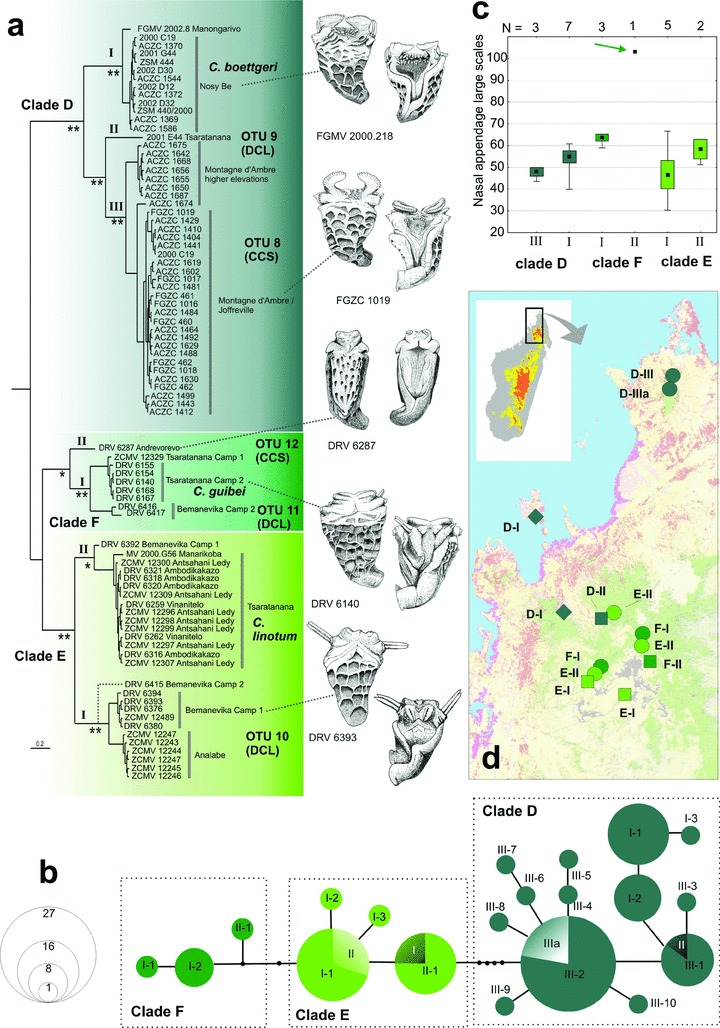
Differentiation among clades in Malagasy chameleons of the *Calumma boettgeri* complex (species of the *C. nasutum* group with occipital lobes: *C. boettgeri, C. guibei, C. linotum*). (a) Majority-rule consensus tree from a Bayesian inference analysis based on a fragment of the *ND2* gene, with exemplary drawings of the hemipenis (asulcal view left, sulcal view right) for major lineages. For convenience, roman numbers were assigned to each lineage. Asterisks denote support from Bayesian posterior probabilities: *PP 0.95–0.98; **PP 0.99–1.0. Abbreviations CCS, UCS, and DCL as in Figure 1. (b) Haplotype network based on a fragment of the nuclear *CMOS* gene (400 bp; 126 haplotype sequences from 63 specimens). Sizes of circles on the left indicate haplotype frequencies. (c) Differentiation of lineages in the number of large scales on the nasal appendage of males (dots, boxes, and whiskers represent mean, standard error, and minimum–maximum values, respectively); note the highly divergent value in lineage II of *C. guibei*. (d) Distribution map of localities sampled for this study (which cover the entire known distribution area of these species). Colors and symbols in map as in Figure 2; bo, gu, and li refer to *C. boettgeri, C. guibei,* and *C. linotum,* with lineage numbers following the abbreviation.

Bayesian inference was carried out on *ND2* sequences from 82 individuals of chameleons of the *C. nasutum* group with distinct occipital lobes, assigned to *C. boettgeri, C. guibei,* and *C. linotum*. The alignment of 389 nucleotides had 211 variable sites of which 153 were parsimony informative. The analysis (Fig. 3a; duplicate sequences not removed) confirmed within this complex the same major lineages as the analysis of the full dataset of 103 unique sequences in Figure 2. Specimens from Montagne d’Ambre in northern Madagascar formed clades corresponding to higher and lower elevations on this mountain, suggesting a possible adaptive diversification. The haplotype network derived from *CMOS* sequences of 63 individual chameleons (duplicate sequences not removed; 126 haplotype sequences after phasing) showed a clear separation of the three species. Samples assigned to clade D were separated by a minimum of five mutational steps from those assigned to the clades E and F, while samples of those two clades were separated from each other by a minimum of three mutational steps (Fig. 3b). Nuclear haplotype sharing was observed between lineages I and II of clade E, and between lineages II and III of clade D, but not between lineages I and II/III of clade D (roman numbers as used in Fig. 3). The haplotype found in the single specimen of lineage II of clade F was separated by 2–3 mutational steps from the haplotypes of lineage I of that clade (*C. guibei*).

### Differentiation in genital and external morphology in the *C. boettgeri* complex

Hemipenes were examined from a total of 16 adult males of the *C. boettgeri* complex. The hemipenes of the three clades representing the complex (clades D, E, and F; Fig. 3a) could be diagnosed by various differences, such as the apical papillary field on the sulcal side present only in clade A and the apical sulcal lobes present only in clade F (Table 1). Several important differences were also found among lineages within clades. The single available male of lineage II in clade F had much smaller and shallower calyces and larger lateral rotulae as compared to the various males examined from lineage I in clade F (*C. guibei*). In clade E, the development of the sulcal lips appears to be different between the two main lineages. And in clade D, the papillary field might be more strongly developed in lineage I (*C. boettgeri*) than in lineage II, although this possible difference requires more in-depth study.

**Table 1:** Summary of hemipenial characters observed within lineages of the *C. nasutum* group characterized by distinct occipital lobes (*C. boettgeri*, *C. guibei*, *C. linotum*). Hemipenial structures that are unique within one species clade are marked with one asterisk. Reliable diagnostic characters distinguishing one lineage from the other lineage within the same clade are in italics and marked with two asterisks

Lineage	Calyces	Sulcal lips	Rotulae	Elongated papillae	Apical papillary field	Apical sulcal lobes
*C.* aff. *boettgeri* clade D, OTU 8	Large, deep (sulcal, asulcal)	Well developed	Large sulcal, small asulcal pair	Laterally between sulcal and asulcal rotulae	*Medially between rotulae	Absent
*C. boettgeri* clade D	Large, deep, (sulcal, asulcal)	Well developed	Large sulcal, small asulcal pair	Laterally between sulcal and asulcal rotulae	*Medially between rotulae, well developed	Absent

*C. guibei* clade F	**Large, deep, angular (sulcal, asulcal)	Well developed	**Two pairs of small rotulae	Laterosulcal position	Absent	*Present
*C.* aff. *guibei* clade F, OTU 12	**Small, rather elongated, shallow (sulcal)	Thin	**Small distal, relatively larger lateral rotulae	Between distal and lateral rotulae	Absent	*Present

*C. linotum* clade I	Large, shallow (sulcal)	**Well developed	*Large sulcal, small distal pair	Between sulcal and apical rotulae	Absent	Absent
*C. linotum* clade I, OTU 10	Large, shallow (sulcal)	**Relatively thin	*Large sulcal, small distal pair	Between sulcal and apical rotulae	Absent	Absent

The shape and number of scales on the rostral appendage in males obviously differs among various species in the *C. nasutum* group (e.g., very long and pointed in *C. gallus* and related species; Fig. 2). Within the *C. boettgeri* complex we noted that the appendage is made of numerous large scales, with very tiny intercalated scales that are difficult to count. In lateral view, 10–47 small intercalated scales were counted in all individuals of clades E and F (*N* = 4 and 7), but they were missing in most individuals of the clade D (*N* = 10; 0–6 scales in eight individuals; 18–24 scales in two individuals). The number of large scales laterally visible on the appendages was 43–70 except in the single individual of lineage II of clade F. This specimen, characterized by a particularly long appendage, had 103 large scales laterally visible on its appendage. Small sample sizes made statistical analyses of these differences impossible. Pending study of additional material, more morphometric and meristic data and detailed hemipenial descriptions will be published in forthcoming taxonomic revisions of these chameleons.

## Discussion

### Mitochondrial lineages and their distribution pattern

Our analyses based on the *ND2* dataset revealed 33 mitochondrial lineages within the *C. nasutum* group that were supported by at least two of the three species delimitation approaches (Fig. 2). These congruent results from multiple species delimitation methods suggest that current taxonomy may greatly underestimate species diversity within this group.

At deeper levels in the tree, we can further distinguish 11 well-supported clades with net *p*-distances in excess of 8.5% between them. These 11 deeper groupings are largely congruent with obvious morphological similarities within these clades and differences between them. Morphologically they roughly agree with current species-level classification: clade A corresponds morphologically to *C. gallus,* clade C to *C. vohibola*, clade D to *C. boettgeri,* clade E to *C. linotum*, clade F to *C. guibei*, clades H and I to *C. fallax* (recovered as monophyletic group but without statistical support), and clades B, G, J, and K to *C. nasutum* (Figs. 2 and S1). For example, similarity of all specimens in clade A to *C. gallus* is based on their relatively long and pointed rostral appendages. Besides the nominal species *C. gallus* from central Madagascar, five OTUs exist within this clade, all of which inhabit the lowland east coast below 900 m (Fig. 2). Males of several of the six OTUs within clade A show differences in their rostral appendage (such as spiny vs. smooth scales). The length, shape, and coloration of rostral appendages are thought to be important visual signals most probably influenced by sexual selection via mate choice by females (e.g., Parcher, 1974; Gehring et al., 2011), although it is uncertain whether the characters showing differences between species are relevant to intraspecific communication.

Clades H and I contain specimens morphologically similar to *C. fallax,* although this species is morphologically poorly defined in comparison with *C. nasutum.* These two clades form a monophyletic group and are mainly distributed at middle and higher elevations. Clade H contains six deep mitochondrial lineages (*C. fallax* and five OTUs), all of which were sampled from mid-elevations (above 800 m a.s.l.; many from above 1200 m a.s.l.). Clade I includes three highly divergent OTUs from the Tsaratanana massif in northern Madagascar. Our analyses found four major clades with morphological resemblance to *C. nasutum*, with a distribution mainly at lower elevations (below 800 m a.s.l.). Clades D–F (the *C. boettgeri* complex) appear to be restricted to northern Madagascar, with a clear elevational zonation: Clade D at low to mid-elevations (0 to ca. 1300 m a.s.l.), clade E at intermediate elevations (1300–1550 m a.s.l.), and clade F at the highest elevations recorded for any species of the *C. nasutum* group (1550–2000 m a.s.l.).

### Candidate species and prospects for future research

Two of the species delimitation approaches used herein rely on sequence thresholds, and we chose a threshold of 6% to define OTUs. This value is not unrealistically low; in other groups of chameleons (i.e., *Bradypodion*, *Kinyongia*) species are characterized by divergences ranging from 5% to 25% for *ND2* (Tolley et al. 2004, 2006, 2011). However, it is clear that using a threshold near the lower limit of divergence values observed between well-established chameleon species implies a risk of overestimating true species numbers. If this evidence were to be uncritically applied to the *C. nasutum* group, and all OTUs accepted as candidate species, our results would suggest a more than fourfold increase in species numbers, from 7 to 33, which probably is an overestimate rather than underestimate of the true number of species in the group.

More detailed (although still preliminary) data from genital morphology, pholidosis, and nucDNA are available for clades D–F forming the *C. boettgeri* complex. We suggest that clades D–F contain three valid nominal species, two unnamed independently evolving units representing species (CCS), but also three examples of lineages that are best interpreted as intraspecific variation (DCL). At present, we have no morphological or genital morphological data available for other clades in the *C. nasutum* group. However, nuclear alleles are not shared among several lineages morphologically similar to *C. nasutum* (Gehring et al., 2011), and our observations (Table 2) indicate various instances of syntopic occurrence of lineages with apparent maintenance of morphological distinctness. This indicates that numerous other mtDNA lineages represent undescribed species, even if the total species diversity in the group will probably stabilize at a number lower than 33.

**Table 2:** Table of localities in which different lineages of the *Calumma nasutum* group were found in direct syntopy (less than 1 km from each other)

Locality	Direct syntopy of	Discreteness of lineages evidenced by
Sahafina	*C. gallus* (clade A)	(1) Mitochondrial differentiation; (2) constant differences in external morphology of males and females
	*C.* aff*. nasutum* (clade G; OTU 16)	

Anosibe An’Ala	*C.* aff*. nasutum* (clade B, OTU 7)	Mitochondrial differentiation
	*C. nasutum* (clade K)	
	*C.* aff*. fallax* (clade H, OTU 20)	

Tarzanville	*C.* aff*. nasutum* (clade B, OTU 7)	(1) Mitochondrial differentiation; (2) constant differences in external morphology of males and females (only between *C.* aff. *gallus* and the two *C*. aff. *nasutum*)
	*C. nasutum* (clade K)	
	*C*. aff. *gallus* (clade A, OTU 3)	

Ambatofotsy	*C.* aff*. nasutum* (clade B, OTU 7)	(1) Mitochondrial differentiation; (2) constant differences in external morphology of males and females
	*C*. aff. *gallus* (clade A, OTU 3)	

Makira forest, Campsite I (Angozongahy)	*C.* aff*. nasutum* (clade A, OTU 6)	(1) Mitochondrial differentiation; (2) differences in external morphology of males
	*C.* aff*. nasutum* (clade G, OTU 14)	

Tsaratanana, Campsite I (Antevialambazaha)	*C. guibei* (clade F)	Morphological differences of males (no *linotum* sequence from this site available, but specimens observed in the wild)
	*C.* aff. *linotum* (clade E, OTU 10)	

Bemanevika forest, Campsite II (near village)	*C. guibei* (clade F)	(1) Mitochondrial differentiation; (2) differentiation in nuclear gene; (3) morphological differences of males
	*C. linotum* (clade E)	

Bemanevika Campsite I	*C. linotum* (clade E)	Only mitochondrial differentiation; no differences in morphology, genital morphology, or nuclear DNA
	*C.* aff. *linotum* (clade E, OTU 10)	

*Calumma nasutum* was assumed to be a widespread species in Madagascar's eastern rainforest band (Brygoo, 1971). Our data show a high genetic subdivision of this and all other species in the *C. nasutum* group, and if a large proportion of the mtDNA lineages indeed represent species, then almost no species of the group would have a range spanning over more than ca. 200 km in a north–south direction, similar to other groups of microendemic organisms in Madagascar (e.g., Wollenberg et al., 2008).

Clearly, our results indicate that a systematic revision and the description of various new species within the *C. nasutum* group are necessary. Such a revision will require intensive work because various important questions remain unclear: (1) monophyly of the *C. nasutum* group cannot be confirmed without the addition of taxa from other closely related species in a phylogenetic context, and including longer DNA sequences from additional genes. (2) Testing with a comprehensive nuclear dataset the monophyly of the *C. boettgeri* complex. These species (*C. boettgeri, C. guibei*, and *C. linotum*) are the only *C. nasutum* group chameleons with distinct occipital lobes, are otherwise morphologically similar to each other, and are restricted to a small area of northern Madagascar (Fig. 3), but the *ND2* phylogeny (Fig. 2) does not support their monophyly. (3) The identity of the available nomina, including several junior synonyms, needs to be clarified. For instance, we here followed the definition of Raxworthy et al. (2008) who used the name *C. linotum* for populations of chameleons from mid-elevations in the Tsaratanana massif, and *C. guibei* for populations at higher elevations in the same regions, but since the type locality of *C. linotum* is unspecified and the holotype of *C. guibei* is a juvenile specimen, this definition requires confirmation. Similarly, the identity of *C. gallus* and *C. nasutum* each require further study (Gehring et al., 2011).

In summary, the present study strongly suggests that the diversity in the Malagasy chameleon fauna is far higher than previously realized. Clearly this understudied fauna merits consideration in conservation efforts, since some of these newly discovered lineages are probably restricted to small geographic areas and face serious danger of extinction due to the rapid ongoing destruction of primary habitats.

## Supporting Information

**Figure S1.** Phylogenetic tree (50% majority rule consensus from a Bayesian inference search in MrBayes)including all 215 *ND2* sequences of Malagasy chameleons of the *Calumma nasutum* group.

**Figure S2.** Detailed ultrametric tree for the Malagasy chameleons of the *Calumma nasutum* group using Bayesian inference analysis with BEAST, a Yule model, and a strict clock.

**Table S1.** List of field numbers and corresponding museum collection numbers of voucher specimens, GPS-based locality information, elevation of locality (m a.s.l.) and GenBank accession numbers of specimens used in this study.

**Table S2.** Estimates of net evolutionary divergence between groups of sequences (*p*-distances).

**Table S3.** Number of singletons and total lineages (including singletons) identified using Species Identifier at different cut-off thresholds.

**Table S4.** GMYC model results under different tree priors and clock models.
