# Supplementary material for: Hiding deep in the trees: discovery of divergent mitochondrial lineages in Malagasy chameleons of the Calumma nasutum group
Source: Ecol Evol. 2012 Jul;2(7):1468–79. doi: 10.1002/ece3.269 (PMC3434913; doi:10.1002/ece3.269)
Supplement: Supplementary file 1 [file ece30002-1468-SD1.doc]

**Hiding deep in the trees: discovery of divergent mitochondrial lineages in Malagasy chameleons of the *Calumma* *nasutum* group**

Philip-Sebastian Gehring, Krystal A. Tolley, Falk Sebastian Eckhardt, Ted M. Townsend, Thomas Ziegler, Fanomezana Ratsoavina, Frank Glaw and Miguel Vences

**Supplementary material**

**Figure SM 1.** Phylogenetic tree (50% majority rule consensus from a Bayesian inference search in MrBayes) including all 215 *ND2* sequences of Malagasy chameleons of the *Calumma nasutum* group. Bayesian posterior probabilities above a threshold of 0.9 are shown; colours illustrate major clades of haplotypes corresponding to Fig. 1. Localities corresponding to the field numbers are given in Table SM 1.


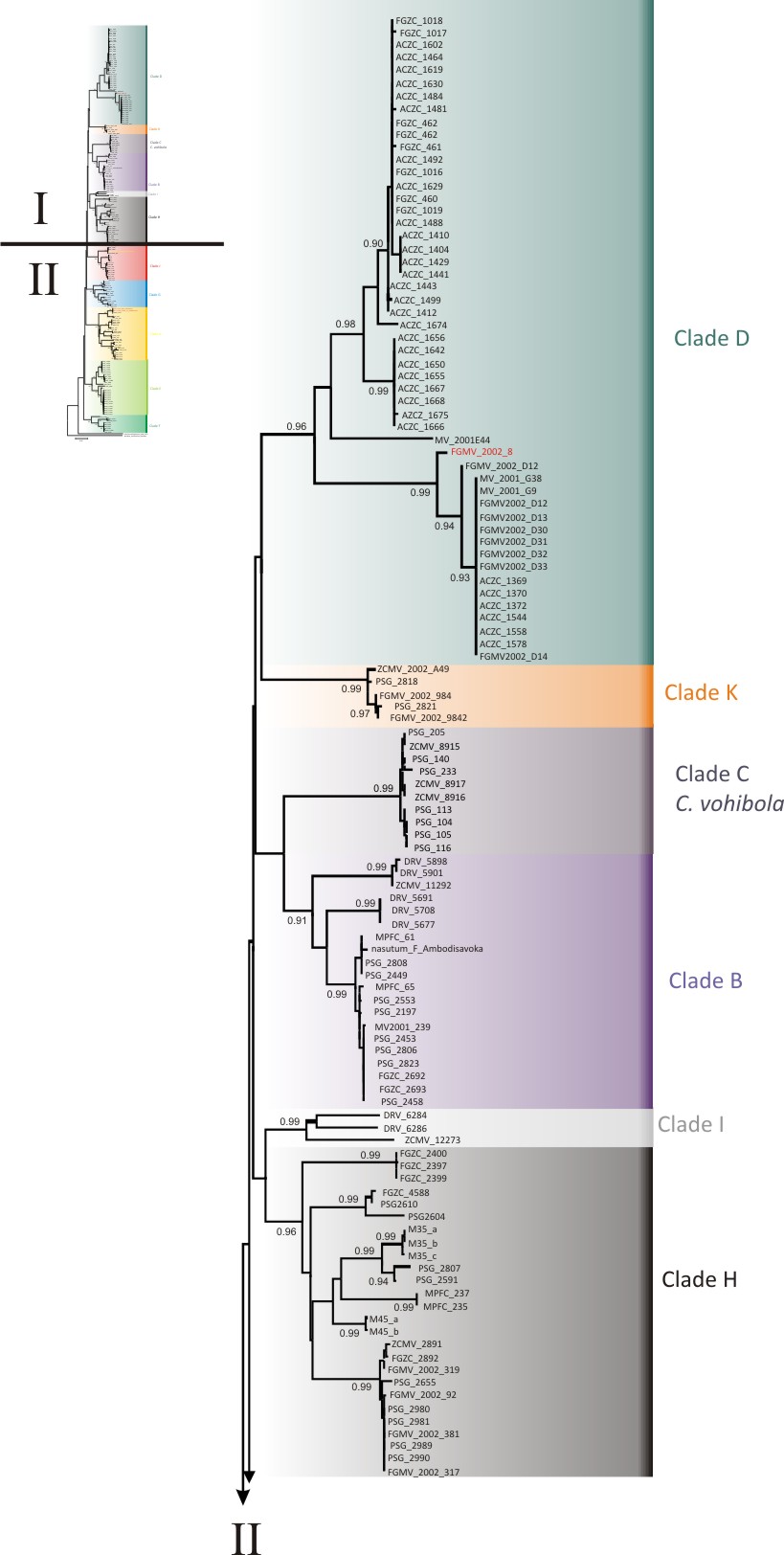


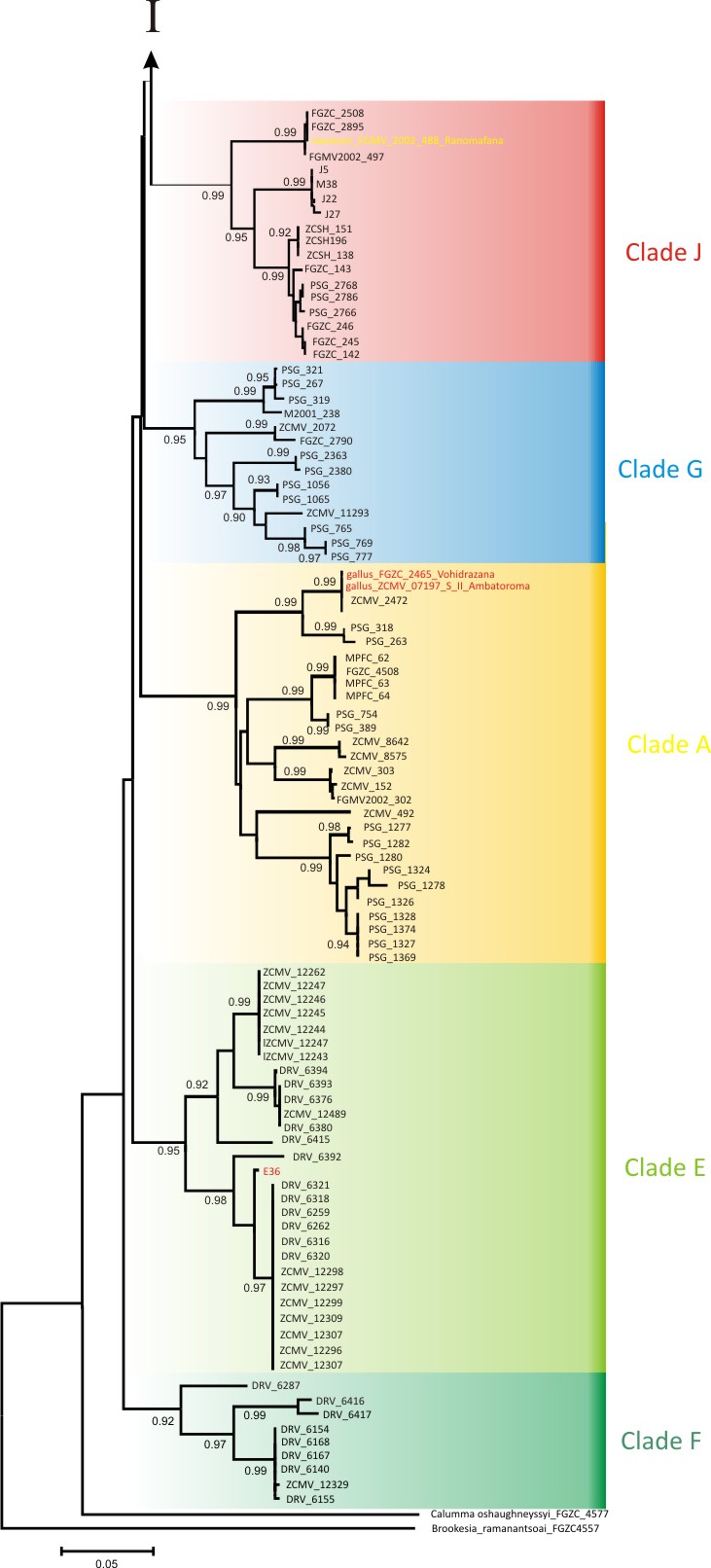


**Figure SM 2.** Detailed ultrametric tree for the Malagasy chameleons of the *Calumma nasutum* group using Bayesian inference analysis with BEAST, a Yule model, and a strict clock. *Calumma oshaughnessyi* and *Brookesia ramanantsoai* were used as outgroup taxa (not shown). Lineages are indicated based on three different clustering methods (net *p*-distances: black dots, SpeciesIdentifier: grey bars, and GMYC clusters: red shading).


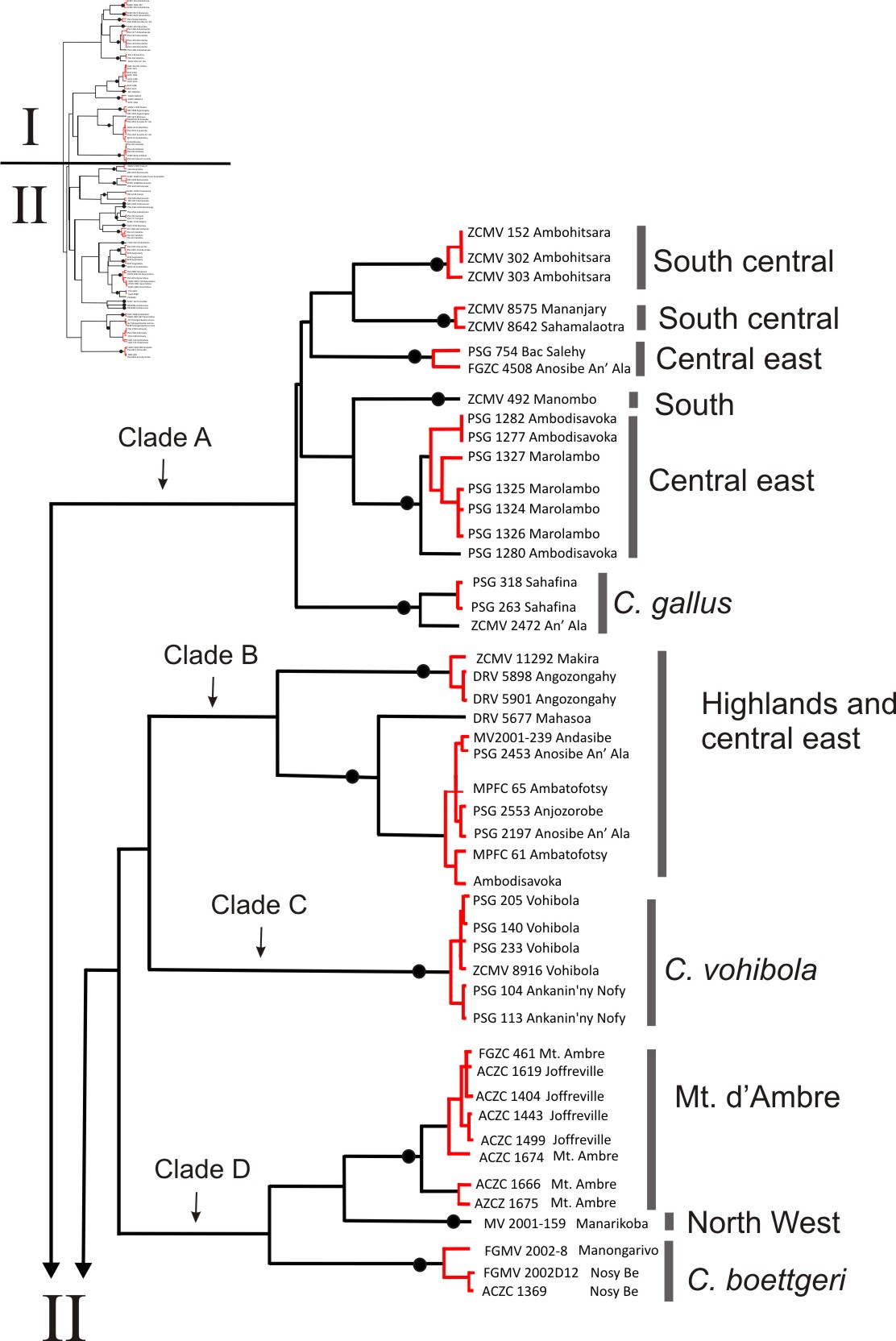


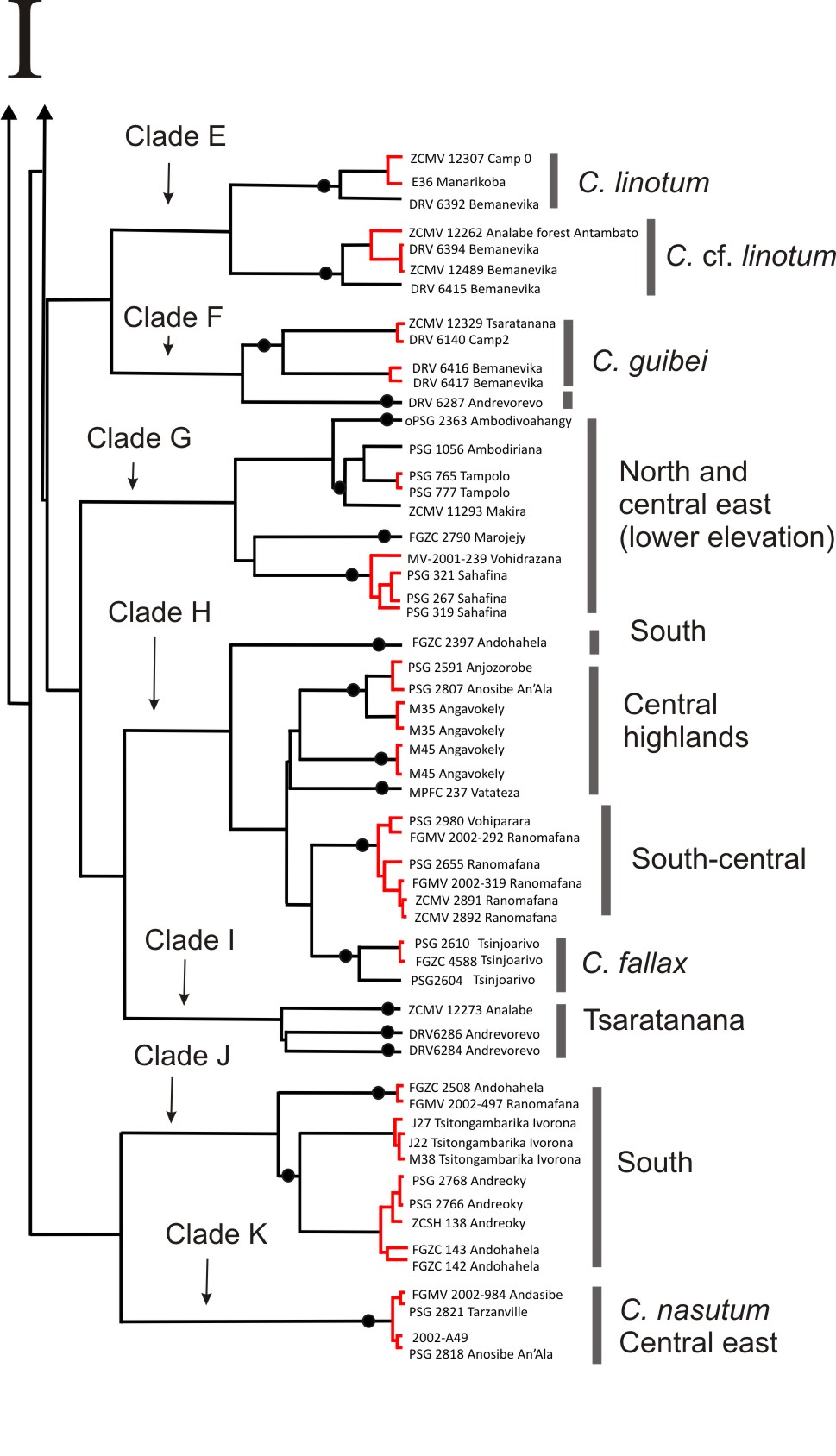


**Table SM 1.** List of field numbers and corresponding museum collection numbers of voucher specimens, GPS based locality information, elevation of locality (m a.s.l.) and GenBank accession numbers of specimens used in this study. Note that some sequences were not submitted to Genbank because they were too short (< 200 bp) and in some other cases, precise locality data are missing. The total number of *ND2* sequences in this list therefore is 199 (which includes all of the 103 unique sequences included in the analysis), whereas a total of 215 sequences were included in the initial screening of diversity in the group.

| **Fieldnumber** | **Genus** | **Species** | **Locality Name** | **Coordinates S** | **Coordinates E** | **Elevation** | **Genbank ND2** | **Genbank Cmos** |
| --- | --- | --- | --- | --- | --- | --- | --- | --- |
| ACZC 1369 | *Calumma* | *boettgeri* | Nosy Be Lokobe periphery | -13.40927778 | 48.30488889 | 26m | JQ734145 | JQ614354 |
| ACZC 1370 | *Calumma* | *boettgeri* | Nosy Be Lokobe periphery | -13.40927778 | 48.30488889 | 26m | JQ734144 | JQ614360 |
| ACZC 1372 | *Calumma* | *boettgeri* | Nosy Be Lokobe periphery | -13.40927778 | 48.30488889 | 26m | JQ734143 |  |
| ACZC 1404 | *Calumma* | cf. *boettgeri* | Joffreville road between Joffreville and the Park entrance | -12.51194444 | 49.19083333 | 943m | JQ734098 |  |
| ACZC 1410 | *Calumma* | cf. *boettgeri* | Joffreville road between Joffreville and the Park entrance | -12.51194444 | 49.19083333 | 943m | JQ734099 | JQ614382 |
| ACZC 1412 | *Calumma* | cf. *boettgeri* | Joffreville | -12.50358333 | 49.19027778 | 873m | JQ734102 | JQ614374 |
| ACZC 1429 | *Calumma* | cf. *boettgeri* | Joffreville | -12.4833 | 49.2 | 626m | JQ734100 | JQ614384 |
| ACZC 1441 | *Calumma* | cf. *boettgeri* | Joffreville | -12.4833 | 49.2 | 626m | JQ734101 |  |
| ACZC 1443 | *Calumma* | cf. *boettgeri* | Joffreville | -12.50358333 | 49.19027778 | 873m | JQ734103 | JQ614385 |
| ACZC 1464 | *Calumma* | cf. *boettgeri* | Joffreville road between Joffreville and the Park entrance | -12.51194444 | 49.19083333 | 943m | JQ734104 |  |
| ACZC 1481 | *Calumma* | cf. *boettgeri* | Joffreville road between Joffreville and the Park entrance | -12.51194444 | 49.19083333 | 943m | JQ734105 | JQ614383 |
| ACZC 1488 | *Calumma* | cf. *boettgeri* | Joffreville road between Joffreville and the Park entrance | -12.51194444 | 49.19083333 | 943m | JQ734107 |  |
| ACZC 1484 | *Calumma* | cf. *boettgeri* | Joffreville road between Joffreville and the Park entrance | -12.51194444 | 49.19083333 | 943m | JQ734106 | JQ614380 |
| ACZC 1492 | *Calumma* | cf. *boettgeri* | Joffreville road between Joffreville and the Park entrance | -12.51194444 | 49.19083333 | 943m | JQ734108 |  |
| ACZC 1499 | *Calumma* | cf. *boettgeri* | Joffreville road between Joffreville and the Park entrance | -12.51194444 | 49.19083333 | 943m | JQ734109 |  |
| ACZC 1544 | *Calumma* | *boettgeri* | Nosy Be | -13.375 | 48.315 | 22m | JQ734142 | JQ614361 |
| ACZC 1558 | *Calumma* | *boettgeri* | Nosy Be | -13.375 | 48.315 | 22m | JQ734141 |  |
| ACZC 1578 | *Calumma* | *boettgeri* | Nosy Be | -13.375 | 48.315 | 22m | JQ734140 |  |
| ACZC 1586 | *Calumma* | cf. *boettgeri* | Nosy Be | -13.375 | 48.315 | 22m |  | JQ614365 |
| ACZC 1602 | *Calumma* | cf. *boettgeri* | Gite d´Etape (Montagne d´Ambre National Park) | -12.52694 | 49.17194 | 1072m | JQ734110 | JQ614376 |
| ACZC 1619 | *Calumma* | cf. *boettgeri* | Gite d´Etape (Montagne d´Ambre National Park) | -12.52694 | 49.17194 | 1072m | JQ734111 | JQ614377 |
| ACZC 1620 | *Calumma* | cf. *boettgeri* | Gite d´Etape (Montagne d´Ambre National Park) | -12.52694 | 49.17194 | 1072m |  | JQ614378 |
| ACZC 1629 | *Calumma* | cf. *boettgeri* | Gite d´Etape (Montagne d´Ambre National Park) | -12.52694 | 49.17194 | 1072m | JQ734112 | JQ614379 |
| ACZC 1630 | *Calumma* | cf. *boettgeri* | Gite d´Etape (Montagne d´Ambre National Park) | -12.52694 | 49.17194 | 1072m | JQ734113 | JQ614375 |
| ACZC 1642 | *Calumma* | cf. *boettgeri* | Westtrail Camp site Lac Maudit (Montagne d´Ambre National Park) | -12.58127778 | 49.14 | 1090m | JQ734122 | JQ614381 |
| ACZC 1650 | *Calumma* | cf. *boettgeri* | Camp site Lac Maudit (Montagne d´Ambre National Park) | -12.58558333 | 49.14569444 | 1306m | JQ734123 |  |
| ACZC 1655 | *Calumma* | cf. *boettgeri* | Camp site Lac Maudit (Montagne d´Ambre National Park) | -12.58558333 | 49.14569444 | 1306m | JQ734124 |  |
| ACZC 1656 | *Calumma* | cf. *boettgeri* | Camp site Lac Maudit (Montagne d´Ambre National Park) | -12.58558333 | 49.14569444 | 1306m | JQ734125 | JQ614388 |
| ACZC 1666 | *Calumma* | cf. *boettgeri* | Camp site Lac Maudit (Montagne d´Ambre National Park) | -12.58558333 | 49.14569444 | 1306m | JQ734126 | JQ614387 |
| ACZC 1667 | *Calumma* | cf. *boettgeri* | Camp site Lac Maudit (Montagne d´Ambre National Park) | -12.58558333 | 49.14569444 | 1306m | JQ734127 | JQ614386 |
| ACZC 1668 | *Calumma* | cf. *boettgeri* | Camp site Lac Maudit (Montagne d´Ambre National Park) | -12.58558333 | 49.14569444 | 1306m | JQ734128 |  |
| ACZC 1674 | *Calumma* | cf. *boettgeri* | Westtrail Camp site Lac Maudit (Montagne d´Ambre National Park) | -12.58127778 | 49.14 | 1090m | JQ734114 |  |
| ACZC 1675 | *Calumma* | cf. *boettgeri* | Westtrail Camp site Lac Maudit (Montagne d´Ambre National Park) | -12.58127778 | 49.14 | 1090m | JQ734097 |  |
| DRV 5677 | *Calumma* | cf. *nasutum* | Mahasoa camp | -17.29769 | 48.70199 | 1032m | JQ734076 |  |
| DRV 5691 | *Calumma* | cf. *nasutum* | Mahasoa camp | -17.29769 | 48.70199 | 1032m | JQ734075 |  |
| DRV 5708 | *Calumma* | cf. *nasutum* | Mahasoa camp | -17.29769 | 48.70199 | 1032m | JQ734074 |  |
| DRV 5898 | *Calumma* | cf. *nasutum* | Angozongahy (camp 1) | -15.43702778 | 49.11861111 | 1009m | JQ734079 |  |
| DRV 5901 | *Calumma* | cf. *nasutum* | Angozongahy (camp 1) | -15.43702778 | 49.11861111 | 1009m | JQ734078 |  |
| DRV 6131 | *Calumma* | *guibei* | Tsaratanana Camp 1 (Antevialambazaha) | -14.17413 | 48.94521 | 1589m |  | JQ614391 |
| DRV 6140 | *Calumma* | *guibei* | Tsaratanana Camp 2 (Matsaborimaika) | -14.15256 | 48.95728 | 2021m | JQ734023 |  |
| DRV 6154 | *Calumma* | *guibei* | Tsaratanana Camp 2 (Matsaborimaika) | -14.15256 | 48.95728 | 2021m | JQ734022 | JQ614389 |
| DRV 6155 | *Calumma* | *guibei* | Tsaratanana Camp 2 (Matsaborimaika) | -14.15256 | 48.95728 | 2021m | JQ734019 |  |
| DRV 6168 | *Calumma* | *guibei* | Tsaratanana Camp 2 (Matsaborimaika) | -14.15256 | 48.95728 | 2021m | JQ734020 | JQ614393 |
| DRV 6167 | *Calumma* | *guibei* | Tsaratanana Camp 2 (Matsaborimaika) | -14.15256 | 48.95728 | 2021m | JQ734021 | JQ614390 |
| DRV 6259 | *Calumma* | *linotum* | Forest Vinanitelo (near Camp 00 - Ambinanitelo) | -14.20972222 | 48.97 | 1280 m | JQ734003 |  |
| DRV 6262 | *Calumma* | *linotum* | Forest Vinanitelo (near Camp 00 - Ambinanitelo) | -14.20972222 | 48.97 | 1280 m | JQ734002 | JQ614410 |
| DRV 6284 | *Calumma* | cf. *fallax* | Andrevorevo A | -14.34644444 | 49.10277778 | 1717m | JQ734072 |  |
| DRV 6286 | *Calumma* | cf. *fallax* | Andrevorevo A | -14.34644444 | 49.10277778 | 1717m | JQ734071 |  |
| DRV 6287 | *Calumma* | cf. *guibei* | Andrevorevo A | -14.34644444 | 49.10277778 | 1717m | JQ734024 | JQ614394 |
| DRV 6316 | *Calumma* | *linotum* | Ambodikakazo camp | -14.20972 | 48.89805556 | 1411m | JQ734001 | JQ614402 |
| DRV 6318 | *Calumma* | *guibei* | Ambodikakazo camp | -14.20972 | 48.89805556 | 1411m | JQ734000 | JQ614398 |
| DRV 6320 | *Calumma* | *linotum* | Ambodikakazo camp | -14.20972 | 48.89805556 | 1411m | JQ733999 | JQ614404 |
| DRV 6321 | *Calumma* | *gubei* | Ambodikakazo camp | -14.20972 | 48.89805556 | 1411m | JQ733998 | JQ614399 |
| DRV 6376 | *Calumma* | cf. *linotum* | Bemanevika Camp 1 (Antsirakala) | -14.43061 | 48.60179 | 1466m | JQ734015 |  |
| DRV 6380 | *Calumma* | cf. *linotum* | Bemanevika Camp 1 (Antsirakala) | -14.43061 | 48.60179 | 1466m | JQ734014 |  |
| DRV 6392 | *Calumma* | *linotum* | Bemanevika Camp 1 (Antsirakala) | -14.43061 | 48.60179 | 1466m | JQ733989 | JQ614396 |
| DRV 6393 | *Calumma* | cf. *linotum* | Bemanevika Camp 1 (Antsirakala) | -14.43061 | 48.60179 | 1466m | JQ734013 |  |
| DRV 6394 | *Calumma* | cf. *linotum* | Bemanevika Camp 1 (Antsirakala) | -14.43061 | 48.60179 | 1466m | JQ734011 | JQ614395 |
| DRV 6414 | *Calumma* | cf. *linotum* | Bemanevika camp 2 | -14.35991 | 48.59022 | 1538m |  | JQ614397 |
| DRV 6415 | *Calumma* | cf. *linotum* | Bemanevika camp 2 | -14.35991 | 48.59022 | 1538m | JQ734004 | JQ614368 |
| DRV 6416 | *Calumma* | *guibei* | Bemanevika camp 2 | -14.35991 | 48.59022 | 1538m | JQ734017 |  |
| DRV 6417 | *Calumma* | *guibei* | Bemanevika camp 2 | -14.35991 | 48.59022 | 1538m | JQ734016 | JQ614392 |
| FG/MV 2002-291 | *Calumma* | cf. *fallax* | between Vohiparara and Ranomafana NP-Entrance | -21.22618 | 47.36960 | 1152m | JQ734067 |  |
| FGMV 2002-292 | *Calumma* | cf. *fallax* | between Vohiparara and Ranomafana NP-Entrance | -21.22618 | 47.36960 | 1152m | JQ734065 |  |
| FGMV 2002-317 | *Calumma* | cf. *fallax* | Ranomafana, Kidonavo bridge | -21.22618 | 47.36960 | 1152m | JQ734066 |  |
| FGMV 2002-319 | *Calumma* | cf. *fallax* | Ranomafana, Vohiparara, Kidonafo Bridge | -21.22618 | 47.36960 | 1152m | JQ734064 |  |
| FGMV 2002-497 | *Calumma* | cf. *nasutum* | Ranomafana NP | -21.26389 | 47.41944 | 983m | JQ734046 |  |
| FGMV 2002-984 | *Calumma* | cf. *nasutum* | Andasibe | -18.9333 | 48.4167 | 937m | JN030482 |  |
| FGMV 2002-D12 | *Calumma* | *boettgeri* | Nosy Be | NA | NA | - | JQ734130 | JQ614356 |
| FGMV 2002-D13 | *Calumma* | *boettgeri* | Nosy Be | NA | NA | - | JQ734138 | JQ614358 |
| FGMV 2002-D14 | *Calumma* | *boettgeri* | Nosy Be | NA | NA | - | JQ734133 | JQ614357 |
| FGMV 2002-D30 | *Calumma* | *boettgeri* | Nosy Be | NA | NA | - | JQ734137 | JQ614362 |
| FGMV 2002-D31 | *Calumma* | *boettgeri* | Nosy Be | NA | NA | - | JQ734136 |  |
| FGMV 2002-D32 | *Calumma* | *boettgeri* | Nosy Be | NA | NA | - | JQ734135 |  |
| FGMV 2002-D33 | *Calumma* | *boettgeri* | Nosy Be | NA | NA | - | JQ734134 | JQ614359 |
| FGZC 142 | *Calumma* | cf. *nasutum* | Andohahela | -24.73833333 | 46.84027778 | 600m | JQ734045 |  |
| FGZC 143 | *Calumma* | cf. *nasutum* | Andohahela | -24.73833333 | 46.84027778 | 600m | JQ734043 |  |
| FGZC 245 | *Calumma* | cf. *nasutum* | Andohahela | -24.73833333 | 46.84027778 | 600m | JQ734044 |  |
| FGZC 246 | *Calumma* | cf. *nasutum* | Andohahela | -24.73833333 | 46.84027778 | 600m | JQ734036 |  |
| FGZC 460 | *Calumma* | cf. *boettgeri* | Montagne d'Ambre | -12.6365 | 49.1637 | 1000m | JQ734119 |  |
| FGZC 461 | *Calumma* | cf. *boettgeri* | Montagne d'Ambre | -12.6365 | 49.1637 | 1000m | JQ734121 | JQ614371 |
| FGZC 462 | *Calumma* | cf. *boettgeri* | Montagne d'Ambre | -12.6365 | 49.1637 | 1000m | JQ734120 | JQ614366 |
| FGZC 1016 | *Calumma* | cf. *boettgeri* | Montagne d'Ambre | -12.51666667 | 49.17666667 | 1050m | JQ734116 | JQ614370 |
| FGZC 1017 | *Calumma* | cf. *boettgeri* | Montagne d'Ambre | -12.51666667 | 49.17666667 | 1050m | JQ734115 | JQ614367 |
| FGZC 1018 | *Calumma* | cf. *boettgeri* | Montagne d'Ambre | -12.51666667 | 49.17666667 | 1050m | JQ734117 |  |
| FGZC 1019 | *Calumma* | cf. *boettgeri* | Montagne d'Ambre | -12.51666667 | 49.17666667 | 1050m | JQ734118 | JQ614369 |
| FGZC 2397 | *Calumma* | cf. *fallax* | Andohahela near camp | -24.54403333 | 46.71411667 | 1548m | JQ734051 |  |
| FGZC 2399 | *Calumma* | cf. *fallax* | Andohahela near camp | -24.54403333 | 46.71411667 | 1548m | JQ734050 |  |
| FGZC 2400 | *Calumma* | cf. *fallax* | Andohahela near camp | -24.54403333 | 46.71411667 | 1548m | JQ734049 |  |
| FGZC 2508 | *Calumma* | cf. *nasutum* | Andohahela near camp | -24.54403333 | 46.71411667 | 1548m | JQ734048 |  |
| FGZC 2692 | *Calumma* | cf. *nasutum* | Vohidrazana | -18.96611 | 48.50972 | 830m | JQ734093 |  |
| FGZC 2693 | *Calumma* | cf. *nasutum* | Vohidrazana | -18.96611 | 48.50972 | 830m | JQ734092 |  |
| FGZC 2790 | *Calumma* | cf. *nasutum* | Marojejy, Camp Mantella | -14.43766667 | 49.77555 | 481m | JQ734030 |  |
| FGZC 2892 | *Calumma* | cf. *fallax* | Ranomafana, Vohiparara, Kidonafo Bridge | -21.22618 | 47.36960 | 1152m | JQ734063 |  |
| FGZC 2895 | *Calumma* | cf. *fallax* | Ambatolahy | -21.2439 | 47.4262 | 919m | JQ734047 |  |
| FGZC 4508 | *Calumma* | cf. *gallus* | Tarzanville forest | -19.3306 | 48.2229 | 847m | JQ734164 |  |
| FGZC 4588 | *Calumma* | *fallax* | Tsinjoarivo | -19.65933333 | 47.73433333 | 1568m | JN030486 |  |
| B8 | *Calumma* | *gallus* | Vohidrazana | -18.96611 | 48.50972 | 830m | JQ734165 |  |
| J5 | *Calumma* | cf. *nasutum* | Tsitongambarika Ivorona | -24.82378 | 46.94914 | 267m |  |  |
| J22 | *Calumma* | cf. *nasutum* | Tsitongambarika Ivorona | -24.82378 | 46.94914 | 267m |  |  |
| J27 | *Calumma* | cf. *nasutum* | Tsitongambarika Ivorona | -24.82378 | 46.94914 | 267m | JQ734035 |  |
| M35 | *Calumma* | cf. *nasutum* | Angavokely | -18.92724 | 47.73680 | 1781m | JQ734056 |  |
| M38 | *Calumma* | cf. *nasutum* | Tsitongambarika Ivorona | -24.82378 | 46.94914 | 267m | JQ734034 |  |
| M45 | *Calumma* | cf. *nasutum* | Angavokely | -18.92724 | 47.73680 | 1781m | JQ734057 |  |
| MPFC 61 | *Calumma* | cf. *nasutum* | Camp Ambatofotsy | -19.54311111 | 48.31652778 | 907m | JQ734083 |  |
| MPFC 62 | *Calumma* | cf. *gallus* | Camp Ambatofotsy | -19.54311111 | 48.31652778 | 907m | JQ734053 |  |
| MPFC 63 | *Calumma* | cf.*gallus* | Camp Ambatofotsy | -19.54311111 | 48.31652778 | 907m | JQ734052 |  |
| MPFC 64 | *Calumma* | cf.*gallus* | Camp Ambatofotsy | -19.54311111 | 48.31652778 | 907m | JQ734084 |  |
| MPFC 65 | *Calumma* | cf. *nasutum* | Camp Ambatofotsy | -19.54311111 | 48.31652778 | 907m | JQ734161 |  |
| MPFC 235 | *Calumma* | cf. *fallax* | Vatateza (Tsinjoarivo camp 3) | -19.7199 | 47.8570 | 1365m | JQ734163 |  |
| MPFC 237 | *Calumma* | cf.*fallax* | Vatateza (Tsinjoarivo camp 3) | -19.7199 | 47.8570 | 1365m | JQ734162 |  |
| MV2001-159 /MV2001-E44 | *Calumma* | *boettgeri* | Manarikoba forest | -14.04222 | 48.76167 | 730 m | JQ734129 |  |
| MV2001-239 | *Calumma* | cf. *nasutum* | Andasibe | -18.9333 | 48.4167 | 937m | JQ734087 |  |
| MV2001-238 | *Calumma* | cf. *nasutum* | Vohidrazana | -18.96611 | 48.50972 | 830m | JQ734032 |  |
| MV2001-G9 | *Calumma* | *boettgeri* | Nosy Be | NA | NA | - | JQ734131 | JQ614364 |
| MV2001-G38 | *Calumma* | *boettgeri* | Nosy Be | NA | NA | - | JQ734132 | JQ614363 |
| MV2001-G56 | *Calumma* | *linotum* | Manarikoba forest | -14.04222 | 48.76167 | 730m | JQ733990 |  |
| PSG 104 | *Calumma* | *vohibola* | Ankanin'ny Nofy, Palmarium | -18.60580556 | 49.21383333 | 5m | JN030474 |  |
| PSG 105 | *Calumma* | *vohibola* | Ankanin'ny Nofy, Palmarium | -18.60580556 | 49.21383333 | 5m | JN030475 |  |
| PSG 113 | *Calumma* | *vohibola* | Ankanin'ny Nofy, Palmarium | -18.60580556 | 49.21383333 | 5m | JN030471 |  |
| PSG 116 | *Calumma* | *vohibola* | Ankanin'ny Nofy, Palmarium | -18.60580556 | 49.21383333 | 5m | JN030476 |  |
| PSG 140 | *Calumma* | *vohibola* | Andranokoitra, Vohibola Forest | -18.5897 | 49.2307 | 19m | JN030477 |  |
| PSG 205 | *Calumma* | *vohibola* | Andranokoitra, Vohibola Forest | -18.5897 | 49.2307 | 9m | JN030473 |  |
| PSG 233 | *Calumma* | *vohibola* | Andranokoitra, Vohibola Forest | -18.5897 | 49.2307 | 9m | JN030478 |  |
| PSG 262 | *Calumma* | *gallus* | Sahafina | -18.8106 | 48.9803 | 56m | JN030488 |  |
| PSG 267 | *Calumma* | cf. *nasutum* | Sahafina | -18.8106 | 48.9803 | 56m | JQ734031 |  |
| PSG 318 | *Calumma* | *gallus* | Sahafina | -18.8106 | 48.9803 | 56m | JN030487 |  |
| PSG 319 | *Calumma* | cf. *nasutum* | Sahafina | -18.8106 | 48.9803 | 56m | JN030484 |  |
| PSG 321 | *Calumma* | cf. *nasutum* | Sahafina | -18.8106 | 48.9803 | 56m | JN030483 |  |
| PSG 754 | *Calumma* | cf. *gallus* | Bac Sahlehy, northern bank of Mangoro | -19.98613889 | 48.78563889 | 3m | JQ734160 |  |
| PSG 757 | *Calumma* | cf. *gallus* | Bac Sahlehy, northern bank of Mangoro | -19.98613889 | 48.78563889 | 3m | JQ734159 |  |
| PSG 765 | *Calumma* | cf. *nasutum* | Tampolo forest | -17.2887 | 49.4116 | 7m | JN030485 |  |
| PSG 769 | *Calumma* | cf. *nasutum* | Tampolo forest | -17.2887 | 49.4116 | 7m | JQ734033 |  |
| PSG 777 | *Calumma* | cf. *nasutum* | Tampolo forest | -17.2887 | 49.4116 | 7m |  |  |
| PSG 1056 | *Calumma* | cf. *nasutum* | Ambodiriana forest | -16.6746 | 49.7028 | 52m | JQ734026 |  |
| PSG 1065 | *Calumma* | cf. *nasutum* | Ambodiriana forest | -16.6746 | 49.7028 | 52m | JQ734025 |  |
| PSG 1277 | *Calumma* | cf. *gallus* | Ambodisavoka | -20.0834 | 48.3044 | 555m | JQ734153 |  |
| PSG 1278 | *Calumma* | cf. *gallus* | Ambodisavoka | -20.0834 | 48.3044 | 555m | JQ734169 |  |
| PSG 1280 | *Calumma* | cf. *gallus* | Ambodisavoka | -20.0834 | 48.3044 | 555m | JQ734158 |  |
| PSG 1282 | *Calumma* | cf. *gallus* | Ambodisavoka | -20.0834 | 48.3044 | 555m | JQ734152 |  |
| PSG 1324 | *Calumma* | cf. *gallus* | Marolambo | -20.0488 | 48.1311 | 409m | JQ734151 |  |
| PSG 1325 | *Calumma* | cf. *gallus* | Marolambo | -20.0488 | 48.1311 | 409m | JQ734080 |  |
| PSG 1326 | *Calumma* | cf. *gallus* | Marolambo | -20.0488 | 48.1311 | 409m | JQ734150 |  |
| PSG 1327 | *Calumma* | cf. *gallus* | Marolambo | -20.0488 | 48.1311 | 409m | JQ734149 |  |
| PSG 1328 | *Calumma* | cf. *gallus* | Marolambo | -20.0488 | 48.1311 | 409m | JQ734148 |  |
| PSG 1369 | *Calumma* | cf. *gallus* | Marolambo | -20.0488 | 48.1311 | 409m | JQ734147 |  |
| PSG 1374 | *Calumma* | cf. *gallus* | Marolambo | -20.0488 | 48.1311 | 409m | JQ734146 |  |
| PSG 2197 | *Calumma* | cf. *nasutum* | Anosibe An'Ala | -19.42801667 | 48.20855 | 594m | JQ734086 |  |
| PSG 2363 | *Calumma* | cf. *nasutum* | Ambodivoahangy | -15.2899 | 49.6203 | 83m | JQ734029 |  |
| PSG 2380 | *Calumma* | cf. *nasutum* | Ambodivoahangy | -15.2899 | 49.6203 | 83m | JQ734028 |  |
| PSG 2449 | *Calumma* | cf. *nasutum* | Anosibe An'Ala | -19.43474167 | 48.20071389 | 636m | JQ734082 |  |
| PSG 2453 | *Calumma* | cf. *nasutum* | Anosibe An'Ala | -19.43474167 | 48.20071389 | 636m | JQ734090 |  |
| PSG 2458 | *Calumma* | cf. *nasutum* | Anosibe An'Ala | -19.43474167 | 48.20071389 | 636m | JQ734091 |  |
| PSG 2553 | *Calumma* | cf. *nasutum* | Anjozorobe / Mananara FL | -18.44976667 | 47.93961944 | 1302m | JQ734085 |  |
| PSG 2591 | *Calumma* | cf. *fallax* | Anjozorobe / Sahaforest Camp | -18.42144444 | 47.93805556 | 1315m | JQ734055 |  |
| PSG 2604 | *Calumma* | *fallax* | Tsinjoarivo Camp 1 | -19.68003333 | 47.77063333 | 1607m | JQ734070 |  |
| PSG 2610 | *Calumma* | *fallax* | Tsinjoarivo, way to Camp 2 | -19.7103 | 47.81824 | 1465m | JQ734069 |  |
| PSG 2655 | *Calumma* | cf. *fallax* | Ranomafana, Ambatolahy street | -21.24387 | 47.42622 | 922m | JQ734068 |  |
| PSG 2766 | *Calumma* | cf. *nasutum* | Andreoky | -24.72577778 | 46.79533333 | 412m | JQ734042 |  |
| PSG 2768 | *Calumma* | cf. *nasutum* | Andreoky | -24.72977778 | 46.79069444 | 314m | JQ734041 |  |
| PSG 2786 | *Calumma* | cf. *nasutum* | Andreoky | -24.72977778 | 46.79069444 | 314m | JQ734040 |  |
| PSG 2806 | *Calumma* | cf. *nasutum* | Anosibe An'Ala | -19.42801667 | 48.20855 | 594m | JQ734089 |  |
| PSG 2807 | *Calumma* | cf. *fallax* | Anosibe An'Ala | -19.42801667 | 48.20855 | 594m | JQ734054 |  |
| PSG 2808 | *Calumma* | cf. *nasutum* | Anosibe An'Ala | -19.42801667 | 48.20855 | 594m | JQ734081 |  |
| PSG 2818 | *Calumma* | cf. *nasutum* | Anosibe An'Ala | -19.42801667 | 48.20855 | 594m | JQ734096 |  |
| PSG 2821 | *Calumma* | cf. *nasutum* | Tarzanville forest | -19.3306 | 48.2229 | 847m | JQ734094 |  |
| PSG 2823 | *Calumma* | cf. *nasutum* | Tarzanville forest | -19.3306 | 48.2229 | 847m | JQ734088 |  |
| PSG 2980 | *Calumma* | cf. *fallax* | Vohiparara | -21.24831667 | 47.40406667 | 981m | JQ734062 |  |
| PSG 2981 | *Calumma* | cf. *fallax* | Vohiparara | -21.24831667 | 47.40406667 | 981m | JQ734061 |  |
| PSG 2989 | *Calumma* | cf. *fallax* | Vohiparara | -21.24831667 | 47.40406667 | 981m | JQ734060 |  |
| PSG 2990 | *Calumma* | cf. *fallax* | Vohiparara | -21.24831667 | 47.40406667 | 981m | JQ734059 |  |
| ZCMV 152 | *Calumma* | cf. *gallus* | Ambohitsara | -21.35718333 | 47.81568333 | 294m | JN030472 |  |
| ZCMV 303 | *Calumma* | cf. *gallus* | Ambohitsara | -21.35718333 | 47.81568333 | 294m | JQ734156 |  |
| ZCMV 492 | *Calumma* | cf. *gallus* | Manombo | -23.01 | 47.73 | 44m | JQ734157 |  |
| ZCMV 2072 | *Calumma* | cf. *nasutum* | Marojejy | -14.43611111 | 49.7675 | 631m |  |  |
| ZCMV 2465 | *Calumma* | *gallus* | An'Ala | -18.93 | 48.47 | 889m | JQ734168 |  |
| ZCMV 2472 | *Calumma* | *gallus* | An'Ala | -18.93 | 48.47 | 889m | JQ734166 |  |
| ZCMV 2891 | *Calumma* | cf. *fallax* | Ranomafana, Kidonavo bridge | -21.22618 | 47.36960 | 1152m | JQ734058 |  |
| ZCMV 7197 | *Calumma* | *gallus* | Ambatoroma | NA | NA | - | JQ734167 |  |
| ZCMV 8575 | *Calumma* | cf. *gallus* | Mananjary | -21.22336667 | 48.29696667 | 33m | JQ734155 |  |
| ZCMV 8642 | *Calumma* | cf. *gallus* | Sahamalotra | NA | NA | - | JQ734154 |  |
| ZCMV 8915 | *Calumma* | *vohibola* | Vohibola | -18.5897 | 49.2307 | 9m | JN030479 |  |
| ZCMV 8916 | *Calumma* | *vohibola* | Vohibola | -18.5897 | 49.2307 | 9m | JN030481 |  |
| ZCMV 8917 | *Calumma* | *vohibola* | Vohibola | -18.5897 | 49.2307 | 9m | JN030480 |  |
| ZCMV 11292 | *Calumma* | cf. *nasutum* | Angozongahy or Ampofoko | -15.43702778 | 49.11861111 | 1009m | JQ734077 |  |
| ZCMV 11293 | *Calumma* | cf. *nasutum* | Angozongahy or Ampofoko | -15.43702778 | 49.11861111 | 1009m | JQ734027 |  |
| ZCMV 12243 | *Calumma* | cf. *linotum* | Analabe forest | -14.50477778 | 48.87597222 | 1361m | JQ734010 | JQ614405 |
| ZCMV 12244 | *Calumma* | cf. *linotum* | Analabe forest | -14.50477778 | 48.87597222 | 1361m | JQ734009 | JQ614403 |
| ZCMV 12245 | *Calumma* | cf. *linotum* | Analabe forest | -14.50477778 | 48.87597222 | 1361m | JQ734008 | JQ614406 |
| ZCMV 12246 | *Calumma* | cf. *linotum* | Analabe forest | -14.50477778 | 48.87597222 | 1361m | JQ734007 | JQ614407 |
| ZCMV 12247 | *Calumma* | cf. *linotum* | Analabe forest, Ambodimanga mountain, near Antambato village | -14.50477778 | 48.87597222 | 1361m | JQ734006 |  |
| ZCMV 12262 | *Calumma* | cf. *linotum* | Analabe forest, Ambodimanga mountain, near Antambato village | -14.50477778 | 48.87597222 | 1361m | JQ734005 |  |
| ZCMV 12273 | *Calumma* | cf. *nasutum* | Analabe forest, Ambodimanga mountain, near Antambato village | -14.50477778 | 48.87597222 | 1361m | JQ734073 |  |
| ZCMV 12296 | *Calumma* | *linotum* | Tsaratanana, forest near Camp 0 (Antsahan'i Ledy) | -14.23319 | 48.98001 | 1207m | JQ733997 |  |
| ZCMV 12297 | *Calumma* | *linotum* | Tsaratanana, forest near Camp 0 (Antsahan'i Ledy) | -14.23319 | 48.98001 | 1207m | JQ733996 |  |
| ZCMV 12298 | *Calumma* | *linotum* | Tsaratanana, forest near Camp 0 (Antsahan'i Ledy) | -14.23319 | 48.98001 | 1207m | JQ733995 |  |
| ZCMV 12299 | *Calumma* | *linotum* | Tsaratanana, forest near Camp 0 (Antsahan'i Ledy) | -14.23319 | 48.98001 | 1207m | JQ733994 |  |
| ZCMV 12300 | *Calumma* | *linotum* | Tsaratanana, forest near Camp 0 (Antsahan'i Ledy) | -14.23319 | 48.98001 | 1207m | JQ733993 | JQ614408 |
| ZCMV 12307 | *Calumma* | *linotum* | Tsaratanana, forest near Camp 0 (Antsahan'i Ledy) | -14.23319 | 48.98001 | 1207m | JQ733992 |  |
| ZCMV 12309 | *Calumma* | *linotum* | Tsaratanana, forest near Camp 0 (Antsahan'i Ledy) | -14.23319 | 48.98001 | 1207m | JQ733991 |  |
| ZCMV 12329 | *Calumma* | *guibei* | Tsaratanana Camp 1 (Antevialambazaha) | -14.17413 | 48.94521 | 1589m | JQ734018 |  |
| ZCMV 12481 | *Calumma* | cf. *linotum* | Analabe forest | -14.50477778 | 48.87597222 | 1361m |  | JQ614409 |
| ZCMV 12489 | *Calumma* | cf. *linotum* | Bemanevika | -14.43061 | 48.60179 | 1466m | JQ734012 | JQ614400 |
| ZCMV 12511 | *Calumma* | cf. *linotum* | Bemanevika | -14.43061 | 48.60179 | 1466m |  | JQ614411 |
| ZCMV 12512 | *Calumma* | cf. *linotum* | Bemanevika | -14.43061 | 48.60179 | 1466m |  | JQ614412 |
| FGMV 2002_A49 | *Calumma* | cf. *nasutum* | Andasibe | NA | NA |  | JQ734095 |  |
| ZCSH 138 | *Calumma* | cf. *nasutum* | Andreoky | -24.72583333 | 46.79533333 | 374m | JQ734039 |  |
| ZCSH 151 | *Calumma* | cf. *nasutum* | Andreoky | -24.72583333 | 46.79533333 | 374m | JQ734038 |  |
| ZCSH 196 | *Calumma* | cf. *nasutum* | Andreoky | -24.72583333 | 46.79533333 | 374m | JQ734037 |  |

**Table SM 2.** Estimates of net evolutionary divergence between groups of sequences (*p*-distances). The number of base differences per site from estimation of net average between groups of sequences are shown. NA: within distance not applicable to clades with only one lineage.

|  |  | **1** | **2** | **3** | **4** | **5** | **6** | **7** | **8** | **9** | **10** | **11** | **12** | **13** | **14** | **15** | **16** | **17** | **18** | **19** | **20** | **21** | **22** | **23** | **24** | **25** | **26** | **27** | **28** | **29** | **30** | **31** | **32** |
| --- | --- | --- | --- | --- | --- | --- | --- | --- | --- | --- | --- | --- | --- | --- | --- | --- | --- | --- | --- | --- | --- | --- | --- | --- | --- | --- | --- | --- | --- | --- | --- | --- | --- |
| **1** | *C. guibei* – clade F | NA |  |  |  |  |  |  |  |  |  |  |  |  |  |  |  |  |  |  |  |  |  |  |  |  |  |  |  |  |  |  |  |
| **2** | *C.* aff. *guibei* – clade F,  OTU 12 | 0.076 | 0.029 |  |  |  |  |  |  |  |  |  |  |  |  |  |  |  |  |  |  |  |  |  |  |  |  |  |  |  |  |  |  |
| **3** | *C.* aff. *boettgeri* – clade D,  OTU 8 | 0.136 | 0.145 | 0.016 |  |  |  |  |  |  |  |  |  |  |  |  |  |  |  |  |  |  |  |  |  |  |  |  |  |  |  |  |  |
| **4** | *C.* aff. *boettgeri* – clade D,  OTU 9 | 0.172 | 0.177 | 0.084 | NA |  |  |  |  |  |  |  |  |  |  |  |  |  |  |  |  |  |  |  |  |  |  |  |  |  |  |  |  |
| **5** | *C. boettgeri* – clade D | 0.180 | 0.202 | 0.118 | 0.139 | 0.003 |  |  |  |  |  |  |  |  |  |  |  |  |  |  |  |  |  |  |  |  |  |  |  |  |  |  |  |
| **6** | *C. linotum* – clade E | 0.137 | 0.128 | 0.127 | 0.154 | 0.152 | 0.025 |  |  |  |  |  |  |  |  |  |  |  |  |  |  |  |  |  |  |  |  |  |  |  |  |  |  |
| **7** | *C. linotum* – clade E,  OTU 10 | 0.125 | 0.112 | 0.143 | 0.169 | 0.169 | 0.091 | 0.006 |  |  |  |  |  |  |  |  |  |  |  |  |  |  |  |  |  |  |  |  |  |  |  |  |  |
| **8** | *C. vohibola* – clade C | 0.152 | 0.142 | 0.145 | 0.150 | 0.193 | 0.124 | 0.146 | 0.005 |  |  |  |  |  |  |  |  |  |  |  |  |  |  |  |  |  |  |  |  |  |  |  |  |
| **9** | *C. gallus* – clade A | 0.145 | 0.131 | 0.155 | 0.181 | 0.169 | 0.148 | 0.144 | 0.167 | 0.024 |  |  |  |  |  |  |  |  |  |  |  |  |  |  |  |  |  |  |  |  |  |  |  |
| **10** | *C. gallus* – clade A,  OTU 3 | 0.138 | 0.124 | 0.163 | 0.173 | 0.190 | 0.145 | 0.149 | 0.168 | 0.089 | 0.013 |  |  |  |  |  |  |  |  |  |  |  |  |  |  |  |  |  |  |  |  |  |  |
| **11** | *C.* aff. *gallus* – clade A,  OTU 2 | 0.170 | 0.146 | 0.173 | 0.187 | 0.191 | 0.155 | 0.153 | 0.181 | 0.079 | 0.083 | 0.004 |  |  |  |  |  |  |  |  |  |  |  |  |  |  |  |  |  |  |  |  |  |
| **12** | *C.* aff. *gallus* – clade A,  OTU 1 | 0.167 | 0.140 | 0.161 | 0.180 | 0.201 | 0.161 | 0.157 | 0.161 | 0.093 | 0.076 | 0.062 | 0.004 |  |  |  |  |  |  |  |  |  |  |  |  |  |  |  |  |  |  |  |  |
| **13** | *C.* aff. *gallus* – clade A,  OTU 4 | 0.170 | 0.154 | 0.154 | 0.168 | 0.204 | 0.179 | 0.173 | 0.182 | 0.097 | 0.089 | 0.096 | 0.106 | NA |  |  |  |  |  |  |  |  |  |  |  |  |  |  |  |  |  |  |  |
| **14** | *C.* aff. *gallus* – clade A,  OTU 5 | 0.160 | 0.155 | 0.156 | 0.174 | 0.190 | 0.178 | 0.163 | 0.174 | 0.092 | 0.095 | 0.093 | 0.096 | 0.084 | 0.015 |  |  |  |  |  |  |  |  |  |  |  |  |  |  |  |  |  |  |
| **15** | *C. nasutum* – clade K | 0.122 | 0.121 | 0.120 | 0.155 | 0.161 | 0.108 | 0.122 | 0.144 | 0.153 | 0.153 | 0.161 | 0.163 | 0.165 | 0.155 | 0.008 |  |  |  |  |  |  |  |  |  |  |  |  |  |  |  |  |  |
| **16** | *C.* aff. *nasutum* – clade B,  OTU 6 | 0.153 | 0.141 | 0.140 | 0.167 | 0.183 | 0.116 | 0.130 | 0.116 | 0.167 | 0.171 | 0.168 | 0.168 | 0.194 | 0.186 | 0.134 | 0.004 |  |  |  |  |  |  |  |  |  |  |  |  |  |  |  |  |
| **17** | *C.*aff. *nasutum* – clade B,  OTU 7 | 0.139 | 0.131 | 0.114 | 0.161 | 0.181 | 0.115 | 0.119 | 0.115 | 0.150 | 0.144 | 0.156 | 0.149 | 0.175 | 0.172 | 0.115 | 0.071 | 0.018 |  |  |  |  |  |  |  |  |  |  |  |  |  |  |  |
| **18** | *C.* aff. *fallax* – clade I,  OTU 22 | 0.157 | 0.140 | 0.141 | 0.167 | 0.200 | 0.140 | 0.134 | 0.143 | 0.175 | 0.169 | 0.178 | 0.159 | 0.180 | 0.168 | 0.149 | 0.144 | 0.136 | NA |  |  |  |  |  |  |  |  |  |  |  |  |  |  |
| **19** | *C.* aff. *fallax* – clade I,  OTU 23 | 0.165 | 0.162 | 0.130 | 0.157 | 0.169 | 0.139 | 0.136 | 0.157 | 0.168 | 0.167 | 0.177 | 0.163 | 0.178 | 0.167 | 0.135 | 0.158 | 0.142 | 0.087 | NA |  |  |  |  |  |  |  |  |  |  |  |  |  |
| **20** | *C.* aff. *fallax* – clade I,  OTU 24 | 0.139 | 0.135 | 0.137 | 0.180 | 0.179 | 0.133 | 0.131 | 0.148 | 0.163 | 0.158 | 0.175 | 0.173 | 0.184 | 0.171 | 0.123 | 0.140 | 0.130 | 0.093 | 0.071 | NA |  |  |  |  |  |  |  |  |  |  |  |  |
| **21** | *C.* aff. *fallax* – clade H,  OTU 17 | 0.144 | 0.147 | 0.144 | 0.167 | 0.177 | 0.149 | 0.146 | 0.147 | 0.161 | 0.162 | 0.156 | 0.169 | 0.167 | 0.159 | 0.144 | 0.147 | 0.140 | 0.133 | 0.139 | 0.127 | 0.000 |  |  |  |  |  |  |  |  |  |  |  |
| **22** | *C. fallax* – clade H | 0.136 | 0.111 | 0.126 | 0.160 | 0.173 | 0.142 | 0.141 | 0.136 | 0.157 | 0.146 | 0.164 | 0.163 | 0.167 | 0.158 | 0.131 | 0.132 | 0.117 | 0.132 | 0.128 | 0.112 | 0.087 | 0.020 |  |  |  |  |  |  |  |  |  |  |
| **23** | *C.*aff. *fallax* – clade H,  OTU 18 | 0.147 | 0.133 | 0.142 | 0.167 | 0.185 | 0.139 | 0.146 | 0.140 | 0.154 | 0.152 | 0.165 | 0.178 | 0.178 | 0.169 | 0.134 | 0.143 | 0.120 | 0.133 | 0.142 | 0.122 | 0.102 | 0.073 | 0.018 |  |  |  |  |  |  |  |  |  |
| **24** | *C.* aff. *fallax* – clade H,  OTU 20 | 0.157 | 0.143 | 0.152 | 0.172 | 0.198 | 0.151 | 0.147 | 0.147 | 0.161 | 0.159 | 0.174 | 0.187 | 0.180 | 0.163 | 0.144 | 0.161 | 0.148 | 0.142 | 0.152 | 0.140 | 0.093 | 0.086 | 0.069 | 0.000 |  |  |  |  |  |  |  |  |
| **25** | *C.* aff. *fallax* – clade H,  OTU 19 | 0.130 | 0.117 | 0.126 | 0.153 | 0.159 | 0.119 | 0.134 | 0.140 | 0.154 | 0.149 | 0.162 | 0.172 | 0.177 | 0.159 | 0.122 | 0.143 | 0.125 | 0.135 | 0.131 | 0.118 | 0.089 | 0.063 | 0.051 | 0.067 | 0.002 |  |  |  |  |  |  |  |
| **26** | *C.* aff. *fallax* – clade H,  OTU 21 | 0.147 | 0.138 | 0.134 | 0.169 | 0.174 | 0.136 | 0.140 | 0.160 | 0.164 | 0.156 | 0.170 | 0.177 | 0.183 | 0.171 | 0.125 | 0.141 | 0.123 | 0.128 | 0.127 | 0.120 | 0.100 | 0.072 | 0.078 | 0.089 | 0.073 | 0.005 |  |  |  |  |  |  |
| **27** | *C.* aff. *nasutum* – clade J,  OTU 25 | 0.158 | 0.135 | 0.139 | 0.165 | 0.172 | 0.125 | 0.157 | 0.152 | 0.158 | 0.172 | 0.177 | 0.163 | 0.179 | 0.171 | 0.135 | 0.158 | 0.146 | 0.149 | 0.144 | 0.155 | 0.157 | 0.143 | 0.145 | 0.161 | 0.137 | 0.148 | 0.001 |  |  |  |  |  |
| **28** | *C.* aff. *nasutum* – clade J,  OTU 26 | 0.145 | 0.135 | 0.132 | 0.157 | 0.158 | 0.115 | 0.140 | 0.129 | 0.138 | 0.142 | 0.159 | 0.143 | 0.159 | 0.153 | 0.116 | 0.136 | 0.118 | 0.133 | 0.119 | 0.125 | 0.135 | 0.119 | 0.134 | 0.138 | 0.116 | 0.129 | 0.060 | 0.028 |  |  |  |  |
| **29** | *C.* aff. *nasutum* – clade G,  OTU 16 | 0.129 | 0.118 | 0.131 | 0.156 | 0.190 | 0.122 | 0.142 | 0.139 | 0.147 | 0.138 | 0.156 | 0.148 | 0.164 | 0.154 | 0.113 | 0.156 | 0.131 | 0.157 | 0.141 | 0.136 | 0.142 | 0.127 | 0.131 | 0.153 | 0.122 | 0.146 | 0.135 | 0.118 | 0.011 |  |  |  |
| **30** | *C.* aff. *nasutum* – clade G,  OTU 15 | 0.135 | 0.129 | 0.138 | 0.169 | 0.170 | 0.101 | 0.120 | 0.131 | 0.132 | 0.113 | 0.129 | 0.124 | 0.145 | 0.142 | 0.128 | 0.130 | 0.112 | 0.134 | 0.121 | 0.107 | 0.112 | 0.119 | 0.118 | 0.141 | 0.108 | 0.130 | 0.129 | 0.109 | 0.069 | 0.000 |  |  |
| **31** | *C.* aff. *nasutum* – clade G,  OTU 13 | 0.131 | 0.144 | 0.140 | 0.169 | 0.186 | 0.131 | 0.146 | 0.160 | 0.171 | 0.150 | 0.168 | 0.172 | 0.185 | 0.165 | 0.139 | 0.150 | 0.142 | 0.162 | 0.155 | 0.136 | 0.134 | 0.138 | 0.134 | 0.156 | 0.127 | 0.154 | 0.173 | 0.148 | 0.097 | 0.081 | 0.002 |  |
| **32** | *C.* aff. *nasutum* – clade G,  OTU 14 | 0.114 | 0.129 | 0.128 | 0.164 | 0.184 | 0.127 | 0.137 | 0.157 | 0.155 | 0.141 | 0.153 | 0.151 | 0.164 | 0.155 | 0.120 | 0.148 | 0.127 | 0.154 | 0.135 | 0.127 | 0.119 | 0.120 | 0.125 | 0.150 | 0.121 | 0.139 | 0.157 | 0.120 | 0.074 | 0.066 | 0.046 | 0.028 |

**Table SM 3.** Number of singletons and total lineages (including singletons) identified using SpeciesIdentifier at different cut-off thresholds.

| **Threshold**  **%** | **Singletons** | **Total** | **#Threshold violations (% of total)** | **Maximum pairwise distance within lineage** |
| --- | --- | --- | --- | --- |
| 2 | 23 | 46 | 3 (6.52%) | 3.35% |
| 3 | 18 | 43 | 1 (2.32%) | 3.35% |
| 4 | 14 | 38 | 3 (7.89%) | 4.88% |
| 5 | 11 | 34 | 3 (8.82%) | 6.16% |
| 6 | 10 | 32 | 3 (9.37%) | 6.91% |
| 7 | 9 | 27 | 3 (11.11%) | 11.46% |
| 8 | 6 | 22 | 5 (22.72%) | 11.56% |
| 9 | 2 | 15 | 8 (53.33%) | 11.66% |
| 10 | 1 | 13 | 6 (46.15%) | 12.18% |

**Table SM 4.** GMYC model results under different tree priors and clock models. Shown are the LRT tests for comparison to a null model (likelihood ratio and p value), number of lineages found (clusters + singletons), and the Chi-square test comparing single and multiple GMYC models. The number of independent lineages hypothesized by each run is given under "Total" and refers to Clusters + Singletons.

| **GMYC model** | **Tree**  **model** | **Clock model** | **LR (p)** | **Clusters** | **Singletons** | **Total** | **Chi-square (p)** |
| --- | --- | --- | --- | --- | --- | --- | --- |
| Single | Yule | Strict | 30.77(***) | 26 | 18 | 44 |  |
| Multiple | Yule | Strict | 35.31 (***) | 27 | 24 | 50 | 4.5 (*ns*) |
|  |  |  |  |  |  |  |  |
| Single | Yule | Relaxed | 28.99(***) | 29 | 21 | 50 |  |
| Multiple | Yule | Relaxed | 32.37(***) | 27 | 23 | 50 | 3.4 (*ns*) |
|  |  |  |  |  |  |  |  |
| Single | Coalescent | Strict | 34.05(***) | 23 | 24 | 47 |  |
| Multiple | Coalescent | Strict | 35.48(***) | 25 | 28 | 53 | 1.4 (*ns*) |
|  |  |  |  |  |  |  |  |
| Single | Coalescent | Relaxed | 35.25(***) | 26 | 18 | 44 |  |
| Multiple | Coalescent | Relaxed | 37.75(***) | 26 | 21 | 47 | 2.5 (*ns*) |
